# Charge-Transfer Enhancement by PEI/MXene Buffer Layer for Boosting Performance of Inverted Perovskite Solar Cells

**DOI:** 10.1021/acsami.6c01017

**Published:** 2026-05-12

**Authors:** João P. F. Assunção, Hugo G. Lemos, Jessica H. H. Rossato, Gabriel L. Nogueira, Mirjana Dimitrievska, Marcos A. Cruz Jr, Silvia L. Fernandes, Sidney A. Lourenço, Frank Nüesch, Carlos F. O. Graeff

**Affiliations:** † School of Sciences, Department of Physics, São Paulo State University (UNESP), Bauru, São Paulo 17033-360, Brazil; ‡ Laboratory of Functional Polymers, 28501 Empa—Swiss Federal Laboratories for Materials Science and Technology, Dübendorf 8600, Switzerland; § Institute of Materials Science and Engineering, Ecole Polytechnique Fédérale de Lausanne (EPFL), Station 12, CH-1015 Lausanne, Switzerland; ∥ Departamento de Geração e Armazenamento de Energia, Instituto Tecnológico de Aeronáutica (ITA), Fortaleza 60415-513, Brazil; ⊥ Nanomaterials Spectroscopy and Imaging Group, Transport at Nanoscale Interfaces Laboratory, Empa—Swiss Federal Laboratories for Materials Science and Technology, Überlandstrasse 129, 8600 Dübendorf, Switzerland; # Oninn—Innovation Center, Belo Horizonte,Minas Gerais 31035-536, Brazil; ¶ Materials Science and Engineering Program (PPGCEM), Federal Technological University of Paraná (UTFPR), Londrina 86036-370, Paraná, Brazil

**Keywords:** polyethylenimine, MXenes, Ti_3_C_2_T_*x*_, buffer layer, perovskite solar cells

## Abstract

Polyethylenimine (PEI) is a promising material for use as a buffer layer between PCBM and the metal cathode of inverted perovskite solar cells (PSCs). Its amine groups generate oriented molecular dipoles, inducing work function shifts at the interfaces, and thereby improving energy alignment. However, the insulator nature of PEI can lead to high series resistance. In this work, we introduced Ti_3_C_2_T_*x*_ MXene into PEI (PEI/MX) to form a solution-processable buffer layer for high-performance *p*–*i*–*n* devices. A set of complementary electrical and spectroscopic techniques confirmed that MXenes optimize PCBM/PEI/MX/Ag interfaces. The improved interfacial features result in suppressed recombination losses, increased charge carrier lifetime, and enhanced extraction of photogenerated charges. These improvements are reflected in the enhanced photovoltaic parameters, particularly the open-circuit voltage and fill factor. Notably, PEI/MX based PSCs show enhanced PCE achieving 22.0 ± 0.8% (23.1% for champion PSC device) when compared to the pure PEI based device (18.9 ± 0.6%). Hence, the results presented in this work indicate that solution-processable PEI/MX is a promising candidate to substitute conventional cathode buffer layer materials.

## Graphical abstract

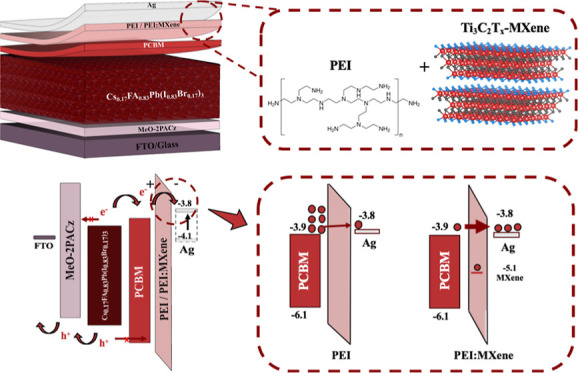

## 1 Introduction

Perovskite solar cells (PSCs) have emerged as a research hotspot in the solar cell field, due to their high-power conversion efficiency (PCE), low-cost fabrication and readiness for large-scale manufacturing. From its various existing architectures, the planar inverted (*p*–*i*–*n*) architecture typically exhibits reduced hysteresis and enhanced stability owing to the use of highly conductive, hydrophobic, and additive-free layers.^1^ In addition, *p*–*i*–*n* devices offer compatibility with flexible substrates and facilitate monolithic integration in tandem solar cells.^2,3^

On the other hand, interfacial defects, poor energy-level alignment along with insufficient charge carrier selectivity cause recombination losses and compromise charge transport and extraction.^4,5^ For instance, although PCBM exhibits high electron mobility and effectively extracts electrons from the perovskite layer, it suffers from an energy-level mismatch with commonly used metal electrodes such as Ag.^6^ This mismatch hinders efficient electron transfer and promotes interfacial recombination, ultimately degrading solar cell performance. Consequently, interfacial engineering strategies aimed at suppressing charge recombination are crucial for improving both device efficiency and long-term stability.^7–9^

Inserting a buffer layer between PCBM and the metal electrode represents a promising interfacial engineering strategy.^10^ In this approach, a cathode buffer layer is used to promote tunneling of photogenerated electrons to the metal electrode due to better energy alignment, while at the same time blocking holes.^11^ Cathode buffer layers such as bathocuproine (BCP)^12–14^ and SnO_*x*_ ^15^ are widely employed, and their thickness can be precisely controlled during physical deposition processes. For example, BCP and SnO_2_ are typically deposited by thermal evaporation and atomic layer deposition (ALD), respectively, enabling in situ thickness monitoring. In addition, BCP can also be deposited via solution-based methods (commonly using isopropanol), which is advantageous from scalability and processability perspectives.

Among solution-processable alternatives, polyethylenimine (PEI) is another strong candidate for use as a buffer layer in an inverted architecture.^16–21^ PEI is a large band gap insulator with primary, secondary and/or tertiary amine groups depending on the polymer molecular structure. These functional groups generate electrical dipole moments, which induce vacuum-level shifts at the interface with adjacent layers, thereby altering their work function (WF).^22,23^ Zhang et al.^24^ for instance used ethoxylated polyethylenimine (PEIE) to decrease the injection barrier between PCBM and the Ag electrode. This surface modification favored the extraction of electrons and resulted in devices with higher *J*_sc_ and PCE.^24^ Similarly, Fang et al.^25^ noticed a reduction in WF of CsPbI_2_Br perovskite and SnO_2_ films following the deposition of branched PEI polymer films on the latter. As expected, the reduced WF with PEI enhanced charge extraction capability of the devices. A thin film of branched PEI was also used by Xie et al.^26^ to minimize the charge injection barrier between PCBM and a silver nanowires (AgNWs) based electrode. In addition to tuning energy levels at the interfaces, the thin PEI layer also mitigated chemical corrosion of the AgNWs resulting in a more uniform and densely packed electrode.

Interestingly, the work function modification induced by PEI is reported to be largely independent of the polymer film thickness.^22^ However, the insulating nature of PEI can cause high series resistance in thicker films. Consequently, device performance is highly sensitive to the PEI layer thickness, with an optimal value typically found around 5 nm.^27,28^ Achieving uniform films within this thickness by solution processing is challenging due to the formation of polymer islands, separated by regions with much thinner film thickness.^22^ Therefore, the application of PEI buffer layers in PSCs relies on achieving uniform polymeric films that simultaneously optimize energy surface and electrical properties.

Notwithstanding the recent advances in cathode buffer layer design, the potential of PEI as an alternative material in PSCs warrants further investigation. In particular, the development of thin PEI films that combine ease of solution processability, tunable surface energy, and favorable electrical properties remains a key challenge. Recent studies have demonstrated the versatile application of Ti_3_C_2_T_*x*_ MXenes in various layers of PSCs, including the perovskite absorber layer,^29,30^ electron transport layers (ETLs)^29,31,32^ and hole transport layers (HTLs).^33^ Additionally, MXenes have been explored as passivating buffer layers.^34,35^ Yakusheva et al.,^34^ for instance, reported the incorporation of MXene as an additive in a solution-processed BCP layer, resulting in devices with improved power conversion efficiency and operational stability. These enhancements are primarily attributed to the high electrical conductivity of MXenes and their ability to suppress ion migration and charge recombination at critical interfaces. Along with their superior electrical conductivity, MXene 2D materials feature high optical transmittance, thermal stability, and favored hydrophilicity.^36^ The functional groups of MXenes can tailor their WF, promoting their use in emerging energy conversion systems such as organic,^33^ dye-sensitized^37^ and perovskite^38^ solar cells.

In this work, we propose blending PEI with highly conductive 2D MXene nanoparticles to increase conductivity and to adjust the effective work function of the cathode. Thus, we applied Ti_3_C_2_T_*x*_ MXene into PEI (PEI/MX) to form a solution-processable hybrid buffer layer for high-performance *p*–*i*–*n* devices. The PEI/MX layer granted a better interface between PCBM and the Ag electrode, increasing charge extraction due to enhanced tunneling of charges promoted by Ti_3_C_2_T_*x*_ and contributing to defect passivation at the interface. The results presented in this work consistently indicate that the incorporation of MXene significantly enhances the functional properties of the PEI buffer layer, making it a strong alternative to conventional materials such as BCP in inverted PSCs.

## 2 Experimental Section

### 2.1 Ti_3_C_2_T_*x*_ MXene Synthesis

The Ti_3_AlC_2_ MAX phase (Sigma-Aldrich) was etched in a 12 M LiF and 9 M HCl solution under constant agitation for 24 h at 25 °C. The mixture was repeatedly washed with high-purity water using centrifugation (3500 rpm, 5 min) until the supernatant reached pH ≈ 5. The MXene was collected by vacuum filtration through a cellulose acetate membrane (0.47 μm pore size) and dried overnight under vacuum at room temperature. Subsequently, 0.5 mg of dried MXene was dispersed in 1 mL of anhydrous isopropyl alcohol (IPA) and was sonicated in an ultrasonic bath for 10 min at 25 °C, yielding a 0.5 mg·mL^–1^ solution.

### 2.2 PEI/MXene Solution Preparation

The polyethylenimine (PEI—Branched, average *M*_w_ ∼ 800, average *M*_n_ ∼ 600, Sigma-Aldrich) solution was prepared in anhydrous IPA at a concentration of 0.2 mg·mL^–1^. Various formulations were fabricated by mixing the MXene and PEI solution (0, 1, 1.5, and 2 wt % of MXene with respect to PEI, designated as PEI/MX).

### 2.3 Perovskite Preparation

Cs_0.17_FA_0.83_Pb­(I_0.83_Br_0.17_)_3_ perovskite solution was obtained by combining iodide- and bromide-based precursor solutions. The iodine-based solution contained 1.2 M of lead­(II) iodide (PbI_2_, TCI Chemicals), 1.0 M of formamidinium lead iodide (FAI, Sigma-Aldrich) and 0.2 M of cesium iodide (CsI, Sigma-Aldrich). The bromide-based solution consisted of 1.2 M of lead­(II) bromide (PbBr_2_, Sigma-Aldrich) and 1.2 M of formamidinium bromide (FABr, Greatcellsolar). Both solutions were prepared in a mixture (4:1 v/v) of anhydrous *N*,*N*-dimethylformamide (DMF, Sigma-Aldrich) and anhydrous dimethyl sulfoxide (DMSO, Sigma-Aldrich) and were stirred overnight at 70 °C. Subsequently, the solutions were combined to obtain a FAPbBr_3_: CsFAPbI_3_ mixture (17:83% v/v) and filtered through a 0.45 μm syringe filter prior to deposition. All steps were carried out in a nitrogen-filled glovebox.

### 2.4 Solar Cell Assembly

Fluorine-doped tin oxide (FTO) coated glasses (7 Ω/sq,^2^ Sigma-Aldrich) were sequentially washed with Extran solution (50% v/v in H_2_O), high-purity water, acetone, and IPA for 20 min each in an ultrasound bath. The dried substrates were treated with UV-Ozone (Ossila) during 15 min. The fabrication of devices was conducted in a nitrogen-filled glovebox. The HTL layer was formed by spin-coating a MeO-2PACz (0.35 mg mL^–1^ in anhydrous IPA, TCI Chemicals) at 3000 rpm for 30 s, followed by thermal annealing at 100 °C for 10 min. The Cs_0.17_FA_0.83_Pb­(I_0.83_Br_0.17_)_3_ perovskite precursor solution was deposited by spin-coating on the HTL (1000 rpm for 5 s and 6000 rpm for 25 s), using anhydrous chlorobenzene (CB) as antisolvent dispensed during the last 5 s of the spinning. After deposition, the perovskite was annealed at 120 °C for 30 min.

The ETL layer was deposited by spin-coating a PCBM (NanoC) solution in CB (20 mg·mL^–1^) at 1000 rpm for 20 s. The PEI and PEI/MX (1, 1.5 and 2%) formulations were spin-coated at 4000 rpm for 60 s. The Ag top electrodes (80 nm) were thermally evaporated. Figure 1a shows a schematic illustration of the device structure.

**Figure 1:**
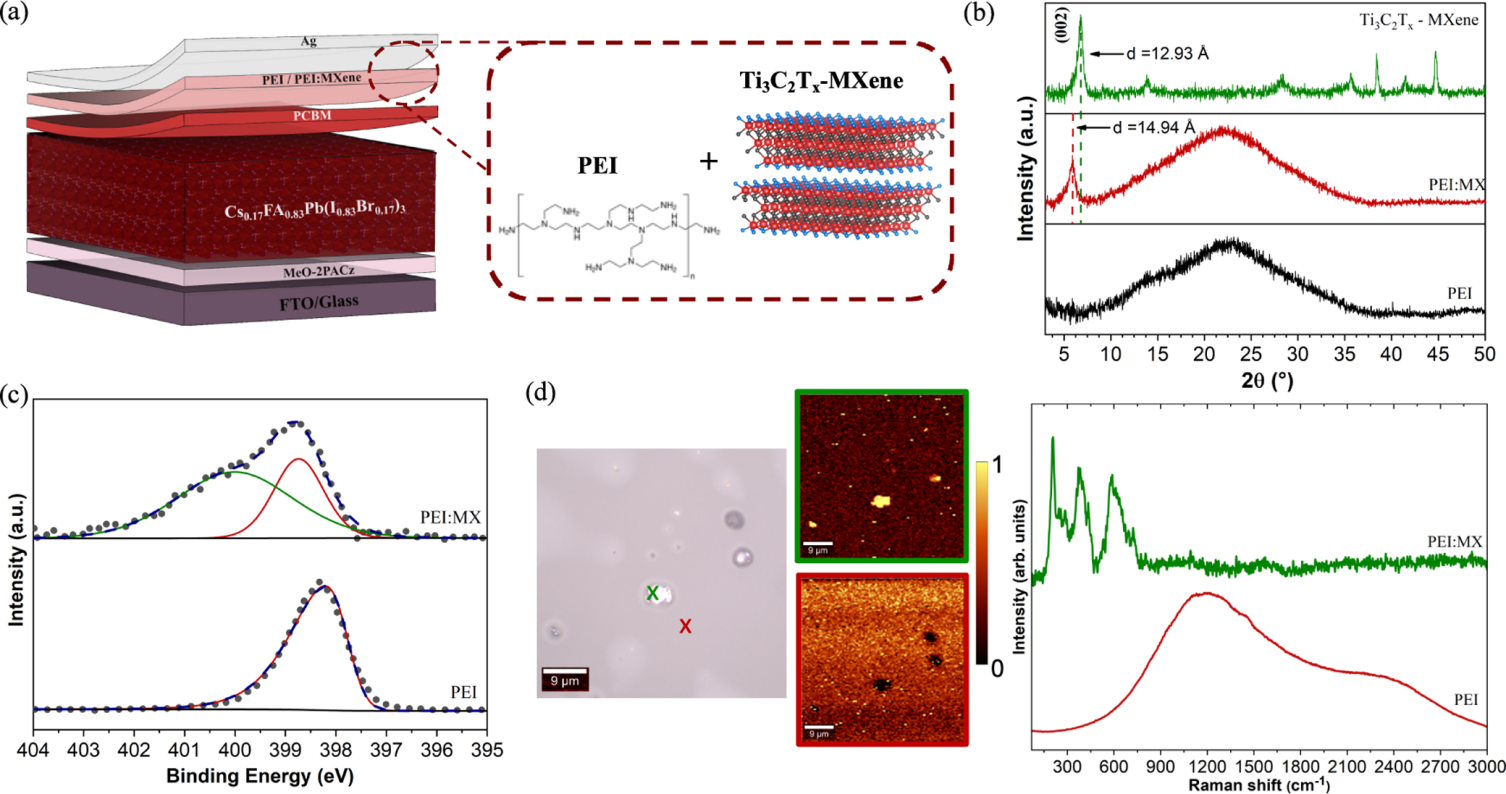
(a) Schematic illustration of the PSC structure and the buffer layer composition. (b) XRD patterns of pure Ti_3_C_2_T_*x*_ MXene, PEI/MX and pure PEI films. (c) XPS N 1s spectra of PEI and PEI/MX films. (d) Optical image, Raman mapping at ∼207 cm^–1^ (green box) and 1200 cm^–1^ (red box) and Raman spectra of PEI and PEI/MX.

### 2.5 Characterization

X-ray diffractograms (XRD) were collected using a Rigaku diffractometer (Cu Kα, λ = 1.54056 Å), model D/MAX-2100/PC, within 2θ from 3° to 50° and an angular step of 0.02°. Field emission scanning electron microcopy (FE-SEM) images were acquired with a ZEISS microscope model GeminiSEM 460. Raman spectra were acquired on a WITec Alpha 300 R confocal microscope in backscattering geometry with 532 nm excitation, focused to ∼1 μm with power of 0.5 mW. Spectra were dispersed by a 300 mm spectrometer with a 600 g/mm grating and detected by a thermoelectrically cooled CCD, with Raman shifts calibrated to the Si peak at ∼520 cm^–1^. X-ray photoelectron spectroscopy (XPS) was measured using an X-ray spectrometer Scienta (Al Kα source, *h*ν = 1486.6 eV), model Omicron ESCA+. The CasaXPS software was used to analyze data. Shirley algorithm was applied for background subtraction. The C 1s peak served as a reference for energy calibration. Atomic force microscopy (AFM) was measured with a Park System AFM microscope, model XE7, in noncontact mode. Contact angle measurements were collected using a contact angle goniometer, from Ossila. Ellipsometry measurements were carried out using an M-2000 spectroscopic ellipsometer (J. A. Woollam Co., Inc.). The Psi (Ψ) and Delta (Δ) parameters were fitted using a B-spline model, with a refractive index (*n*) of 1.529 as provided by the material supplier. Kelvin probe (KP) and surface photovoltage (SPV) measurements were performed using a KP020 system (KP Technology), with sample illumination provided by a 150 W Fiber-Lite DC950 illuminator (Dolan–Jenner).

Current density versus voltage (*J*–*V*) curves of PSCs were measured using a Keithley 2400 source measurement and a solar simulator (Newport, 94023A-U) under simulated sunlight at AM 1.5G illumination (100 mW cm^2^). The calibration of the sunlight intensity of the solar simulator was certified with a silicon solar cell. External quantum efficiency (EQE) spectra were performed using a PTS-2-QE System, from Sciencetech. A PAIOS system from Fluxim was used for all additional electrical characterizations: dark *J*–*V* curves, transient photocurrent (TPC), charge extraction by a linearly increasing voltage (photo-CELIV), light Intensity CELIV and electrical impedance spectroscopy (EIS). Steady-state photoluminescence (PL) was performed using a 405 nm diode laser, as the excitation source, and a BRC112E-USB-VIS/NIR (Edmund Optics) mini-spectrometer to detect emission light. Time-resolved PL (TRPL) spectroscopy was measured using a 470 nm picosecond pulsed diode laser and a FluoTime 200 spectrometer (PicoQuant). Devices’ stability was evaluated by maximum power point tracking (MPPT) measurements of encapsulated PSCs following the ISOS-L-2 protocol.

## 3 Results and Discussion

### 3.1 Film Characterization

The successful exfoliation of the MAX phase to Ti_3_C_2_T_*x*_ MXene was confirmed by XRD analysis (Figure S1). The MAX phase diffractogram shows characteristic Bragg refractions of the lamellar carbide crystalline lattice, whereas the Ti_3_C_2_T_*x*_ have reduced and widened peaks due to the delamination of the crystalline structure. Additionally, the (002) diffraction peak shifted markedly from 9.7° in the MAX phase to 6.6° in the MXene, indicating an increased interlayer spacing caused by the intercalation of surface functional groups (–OH, –O, and –F).^39^ FE-SEM images of MXene flakes are shown in Figure S2a–d. The exfoliation of the MAX phase resulted in large flakes with average dimensions of 4.5 ± 2.0 μm, exhibiting wrinkled and crumpled edges as a result of the acid-etching process. These features indicate successful exfoliation of the precursor MAX phase into few-layer MXene sheets.

Structural and spectroscopic analyses were carried out to assess the presence of Ti_3_C_2_T_*x*_ MXene into PEI. XRD patterns of PEI, PEI/MX and MXene films are shown in Figure 1b. Pure PEI diffractograms show a broad peak at 2θ ≈ 22°, which indicates the amorphous nature of the polymer. PEI/MX exhibits an emerging peak at 2θ ≈ 5.8° which is assigned to the (002) plane of Ti_3_C_2_T_*x*_. Noticeably, PEI/MX exhibits a broader and further shifted (002) plane peak when compared to the as prepared MXene pattern. The calculated interlayer distance of Ti_3_C_2_T_*x*_ sheets expanded from 12.93 Å (Ti_3_C_2_T_*x*_) to 14.94 Å (PEI/MX), evidencing an intercalation of the polymeric PEI chains between MXene sheets. This phenomenon may be induced by the negatively charged functional groups on the surface of Ti_3_C_2_T_*x*_ sheets which can interact via hydrogen bonding with the amine groups present in the PEI structure.

XPS measurements were carried out to verify the interaction of PEI and MXene. High-resolution N 1s spectrum of PEI (Figure 1c) exhibits a single peak at 398.5 eV related to neutral amine groups (–NH_2_), as previously reported.^40^ In contrast, the high-resolution N 1s spectrum of PEI/MX (Figure 1c) reveals an additional peak at 400.5 eV, which is attributed to protonated amine groups (–NH_3_^+^)^41^ due to the interaction between PEI and the –OH functional groups of MXenes. These results, associated with the further spacing of the MXene sheets, confirm that the material is encapsulated within the PEI polymer matrix.

The distribution of the MXenes throughout the PEI/MX film was evaluated by Raman imaging, as shown in Figure 1d. Raman spectra were measured over a 50 × 50 μm area. Figure 1d compares spectra from two regions enriched in MXene and PEI, respectively. Raman spectrum of Ti_3_C_2_T_*x*_ exhibits peaks at 207, 377, and 600 cm^–1^ related to the A_1g_ (Ti, C and T_*x*_) vibrational modes, whereas a small peak at 730 cm^–1^ is identified as the out-of-plane A_1g_ mode of C.^42^ Raman spectrum of PEI shows a large band centered at ∼1200 cm^–1^ likely containing contributions of both C–N stretching vibrations (1000–1200 cm^–1^) and CH_2_ wagging and twisting modes (1200–1400 cm^–1^).^43^ In addition, the small shoulder at ∼1450 cm^–1^ is attributed to CH_2_ scissoring and NH in-plane bending vibrations of PEI chains.^44^ Figure 1d displays the mapping of MXene (micrograph with green-square highlighted) and PEI (micrograph with red-square highlighted) based on the peaks at ∼207 and 1200 cm^–1^, respectively, permitting a direct image of Ti_3_C_2_T_*x*_ distribution on the sample. Raman mapping indicates favored distribution of the 2D material throughout the polymeric film resulting in a homogeneous buffer layer. Most visible 2D flakes have sizes below 3 μm. It is important to mention that due to limiting resolution, smaller particles might be present but not seen.

The uniform distribution of Ti_3_C_2_T_*x*_ within the PEI matrix ensured that the incorporation of MXene did not induce any significant changes in the morphological or topographic features of the PEI/MX films. AFM images of PEI and PEI/MX (1.5%) on top of ITO/SAM/perovskite/PCBM stacks are shown in Figure 2. No considerable changes in the root-mean-square roughness (RMS ≈ 9 nm) was noticed after incorporation of MXene into PEI films. To confirm the thin nature of PEI layer, its thickness was estimated by ellipsometry measurements on a Si substrate. Fitting of Ψ and Δ parameters showed a thickness of 2 ± 1 nm, with a root-mean-square (RMS) error of 0.755.

**Figure 2:**
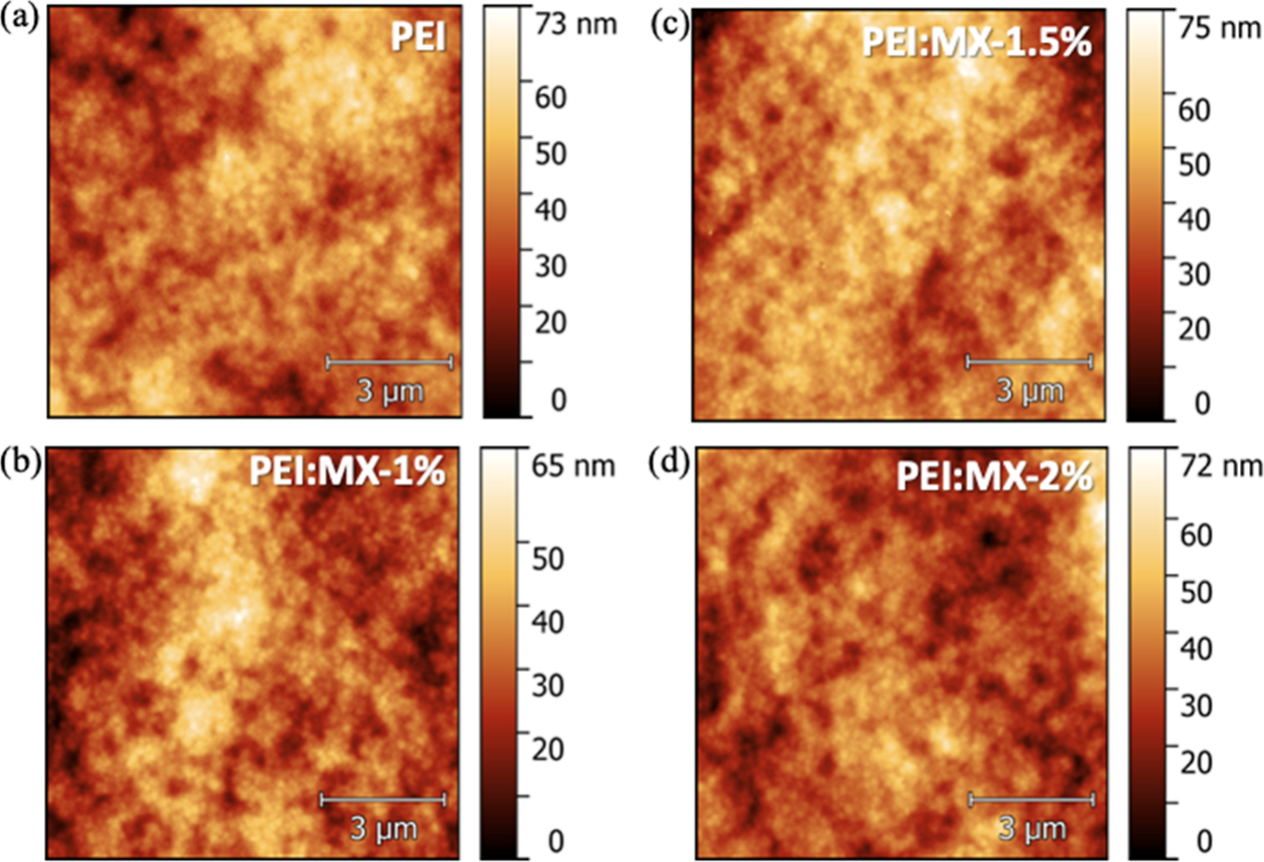
AFM images of perovskite/PCBM films coated with (a) PEI, (b) PEI/MX-1%, (c) PEI/MX-1.5% and (d) PEI/MX-2%.

The hydrophobicity of these films was assessed by contact angle (CA) measurements. The images of the water droplets on top of PEI and PEI/MX (1, 1.5 and 2%) and their respective calculated angles are shown in Figure S3. PEI (Figure S3a) buffer layer showed a CA of 65°, which decreased to 62° (PEI/MX-1%, Figure S3b) and 60° (PEI/MX-1.5%, Figure S3c). The increased hydrophilicity after addition of MXenes is related to the presence of the characteristic polar groups of Ti_3_C_2_T_*x*_. In contrast, PEI/MX-2% (Figure S3d) has an increased CA value of 67° likely due to the enhanced agglomeration and of the 2D material. It is noteworthy that the contact-angle variation between pure PEI and PEI/MX composites is very small, suggesting that the buffer layer retains a hydrophobic tendency that serves as a protective capping layer for the device.

### 3.2 PSCs Performance and Recombination Dynamics

To assess the effects of the PEI/MX buffer layer on the PSCs performance, we fabricated devices with the architecture FTO/SAM/Cs_0.17_FA_0.83_Pb­(I_0.83_Br_0.17_)/PCBM/(PEI/MX)/Ag. For comparison, devices with pure PEI were also assembled. Preliminary, different PEI solution concentrations were systematically evaluated. Based on these optimization experiments, a concentration of 0.2 mg mL^–1^ for PEI was selected for this study, as it yielded the best overall device performance. The box plot analysis (Figure S4) confirms that this concentration provides improved efficiency and reproducibility compared to the other tested conditions. Figure 1a presents the stack structure of PSCs containing PEI and PEI/MX buffer layers, in which PEI and Ti_3_C_2_T_*x*_ MXene structures are highlighted, respectively. *J*–*V* curves in the reverse scan under 1 sun illumination (AM 1.5G, 100 mW cm^–2^) of the best performing devices are shown in Figure 3a. The photovoltaic parameters are summarized in Table 1 and the device statistics are shown in the box plot (Figure S5). A good reproducibility of the PSCs is observed as seen by the small deviations in the values of short-circuit current density (*J*_SC_), open-circuit voltage (*V*_OC_), fill factor (FF) and PCE.

**Figure 3:**
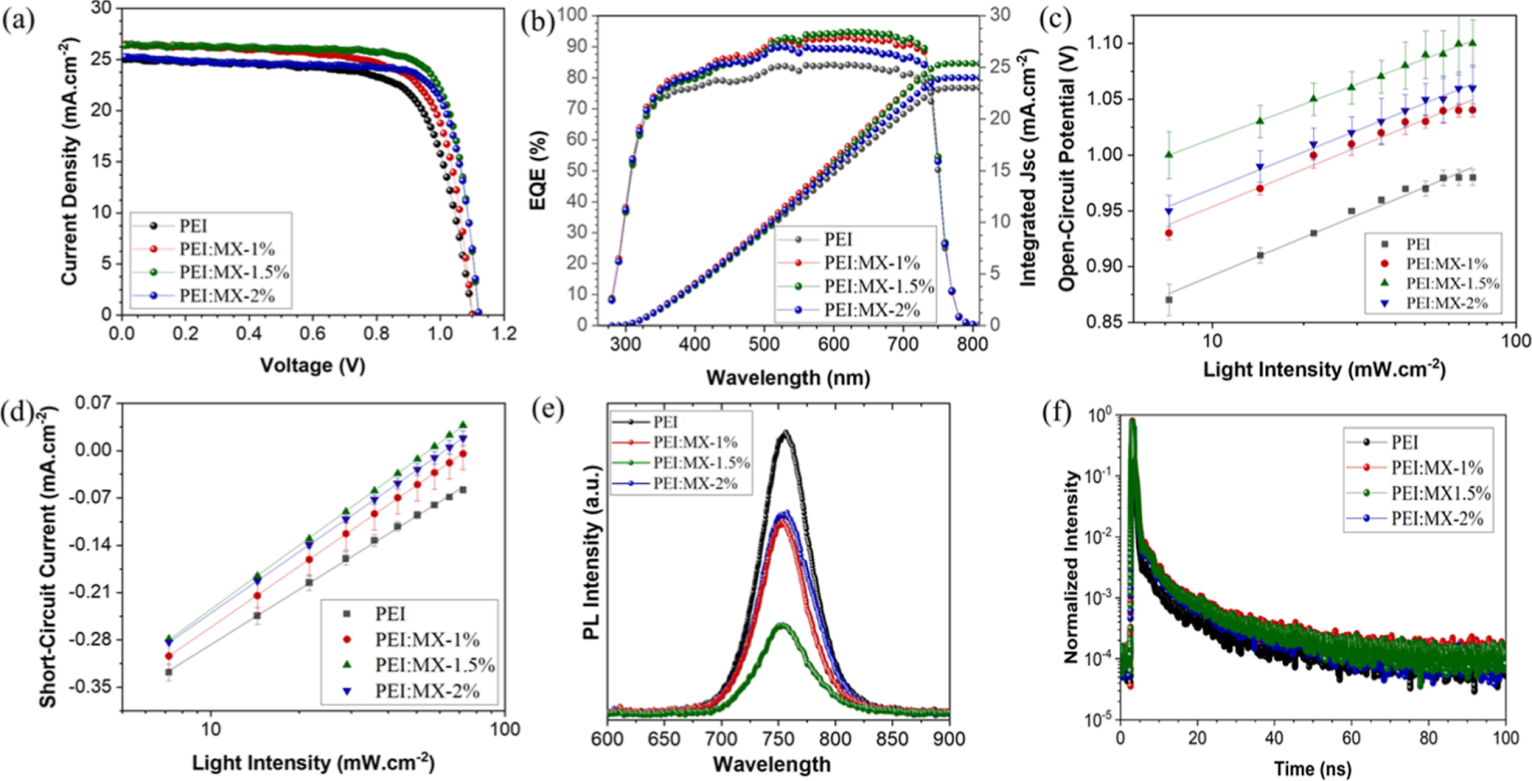
(a) Current density vs voltage (*J*–*V*) curves of PEI and PEI/MX­(1, 1.5 and 2%) PSCs. (b) EQE and integrated *J*_sc_ of PEI and PEI/MX­(1, 1.5 and 2%) devices. Irradiation intensity dependent *V*_oc_ (c) and *J*_sc_ (d) of PEI and PEI/MX­(1, 1.5 and 2%) PSCs. The error bars represent the statistics from 7 different devices. (e) PL and (f) TRPL of perovskite/PCBM/PEI and PEI/MX­(1, 1.5 and 2%).

**Table 1:** Photovoltaic Parameters of the PEI and PEI/MX PSCs under AM 1.5G (100 mW cm^–2^)

device		*J*_sc_ (mA cm^–2^)^a^	*V*_oc_ (V)^a^	FF (%)^a^	PCE (%)^a^	HI
PEI	**Rev**	24.3 ± 0.8 (25.0)	1.10 ± 0.02 (1.10)	70.8 ± 3.0 (71.4)	18.9 ± 0.6 (19.6)	4.21 ± 5.16
	**Fwd**	24.2 ± 1.07 (25.1)	1.10 ± 0.02 (1.11)	72.8 ± 3.5 (71.2)	19.4 ± 1.2 (19.8)	
PEI/MX-1%	**Rev**	24.4 ± 1.1 (26.5)	1.10 ± 0.02 (1.10)	73.8 ± 1.9 (72.5)	19.8 ± 0.9 (21.1)	2.84 ± 2.37
	**Fwd**	24.4 ± 1.0 (26.5)	1.10 ± 0.02 (1.11)	71.0 ± 6.0 (75.1)	19.5 ± 1.5 (22.1)	
PEI/MX-1.5%	**Rev**	25.4 ± 0.9 (26.4)	1.12 ± 0.01 (1.12)	77.2 ± 1.3 (78.1)	22.0 ± 0.8 (23.1)	2.22 ± 1.72
	**Fwd**	25.0 ± 0.8 (25.9)	1.12 ± 0.01 (1.12)	77.0 ± 1.6 (79.1)	21.5 ± 0.8 (22.9)	
PEI/MX-2%	**Rev**	25.0 ± 0.8 (25.3)	1.10 ± 0,02 (1.11)	73.2 ± 2.6 (77.0)	20.2 ± 1.0 (21.8)	3.03 ± 1.97
	**Fwd**	24.8 ± 0.9 (24.3)	1.11 ± 0.01 (1.12)	73.3 ± 2.4 (74.9)	20.1 ± 0.9 (20.3)	

a Results are reported as the mean ± standard deviation of at least seven PSCs for each formulation. The applied bias voltage was measured in both reverse (Rev) and forward (Fwd) scans. The value in parentheses corresponds to the highest measured for each configuration.

A crescent increase in the cell performance was observed with the addition of MXene into PEI buffer layer. Even for the devices with the lowest MXene content, PEI/MX-1%, PCE values showed an increase from 18.9 ± 0.6 (PEI) to 19.8 ± 0.9%. PEI/MX-1.5% based PSCs show the highest values of *J*_SC_ and *V*_OC_ of 25.4 ± 0.9 mA cm^–2^ and 1.12 ± 0.01 V, respectively. The enhanced *V*_OC_ and FF (77.2 ± 1.3) of PEI/MX-1.5% PSCs suggest decreased recombination losses, while the increased *J*_SC_ indicates more efficient charge extraction. The overall improvements led to an enhanced PCE of 22.0 ± 0.8% (23.1% for the best performing device), which is 17% higher than the PEI based devices. However, additional content of MXene results in a decrease of the photovoltaic parameters, as shown for PEI/MX-2% in Table 1.

External quantum efficiency (EQE) spectra and the integrated *J*_sc_ of the PSCs are shown in Figure 3b. Following the trend in performance, the EQE spectra shows the highest values for PEI/MX-1.5% PSCs, with 94.7% of quantum efficiency, while PEI devices showed the lowest efficiency with 84.1%. The integrated current densities were obtained by convoluting the EQE spectra with the AM1.5 solar irradiation spectrum. The calculated values are similar to *J*_SC_ obtained from *J*–*V* curves (Table 1), which corresponds to 25.38 mA cm^–2^ for PEI/MX-1.5% and 23.02 mA cm^–2^ for PEI-based PSCs.

Hysteresis indexes (HI) were estimated from eq S1 and are shown in Table 1. The incorporation of MXene (PEI/MX-1.5%) reduced the hysteresis index (HI) from 4.2% (PEI) to 2.2%. The hysteresis behavior in PSCs is generally attributed to multiple interrelated processes, including ion migration and vacancy redistribution within the perovskite layer, interfacial charge trapping, imbalance in electron and hole extraction, and trap-assisted recombination at grain boundaries or at the interfaces with charge transport layers.^35^ The incorporation of MXene into the PEI interfacial layer enhances electrical conductivity and promotes more efficient electron extraction. This improved interfacial charge transport reduces charge accumulation and mitigates interfacial trapping, ultimately contributing to the suppression of hysteresis.

To confirm our hypotheses of better charge extraction and suppression of recombination losses for PEI/MX based devices, we performed a variety of electrical and optoelectronic characterization. First, we calculated the series (*R*_S_) and shunt resistances (*R*_SH_) of the devices from the dark *J*–*V* curves (Figure S6). The corresponding values of *R*_S_ and *R*_SH_ are shown in Table 2. The *R*_S_ progressively decreases with MXene addition, dropping to 9.1 Ω (PEI/MX-2%) against 32.8 Ω for pure PEI. The *R*_S_ controls the injection and extraction of charge carriers in the devices. Therefore, a lower *R*_S_ indicates better charge injection, which aligns with the photovoltaic performance shown in Table 1. In addition, PEI/MX-1.5% shows the highest R_SH_ (3.8 × 10^6^ Ω) when compared to PEI (3.4 × 10^6^ Ω). The *R*_SH_ is primarily associated with “leakage currents” within the device. Therefore, an increase in *R*_SH_ is linked to a suppression in leakage current and, as a result, an improvement in fill factor (FF) and overall PSC performance.^45^ The reduced FF (73 ± 3%) for PEI/MX-2% suggests poorer carrier extraction and increased recombination indicating a degradation of the tunneling barrier. The reduction in *R*_SH_ at higher MXene loadings (PEI/MX-2%) likely arises from the formation of percolation pathways through the PEI layer that establish electrical contact with the silver electrode. As the MXene network becomes denser, it can extend laterally across the interlayer, forming low-resistance shunt channels that promote leakage currents. Furthermore, MXene nanosheets tend to restack, leading to film nonuniformities and aggregate-induced trap states that enhance interfacial recombination.

**Table 2:** Series Resistance (*R*_s_), Shunt Resistance (*R*_sh_), Ideality Factor (*n*), Power Parameter of *J*_sc_ Ligh Dependent Measurement (α), Trap Density (*N*_t_) Parameters of PEI and PEI/MX­(1, 1.5 and 2%) PSCs

device	*R*_S_ (Ω)	*R*_SH_ (Ω)	*n*	α	*N*_t_ (cm^–3^)
PEI	32.8	3.4 × 10^6^	1.90	0.85	5.01 × 10^17^
PEI/MX-1%	22.3	2.8 × 10^6^	1.88	0.93	4.55 × 10^17^
PEI/MX-1.5%	15.8	3.8 × 10^6^	1.72	0.98	4.15 × 10^17^
PEI/MX-2%	9.1	1.1 × 10^6^	1.84	0.94	4.83 × 10^17^

The recombination kinetics of the PSCs were evaluated by different methods. We monitored the *V*_oc_ of the devices under various light intensities to estimate the ideality factor (*n*) according to eq S2. Figure 3c displays the measured *V*_oc_ at different light intensities, and the calculated values of n are shown in Table 2. In general, values of *n* = 1 indicate the predominance of second-order charge-carrier radiative recombination, whereas *n* = 2 denotes first-order trap-assisted nonradiative recombination.^46^ Under in-operando light conditions, both mechanisms can coexist in the PSC. The ideality factor of PEI-based devices suggests that trap-assisted nonradiative recombination dominates, likely originating at the interface. This recombination is reduced upon the introduction of MXenes. The reduced values of n for PEI/MX-based devices are reflected in the corresponding higher *V*_oc_, as shown in Table 1. We also measured the *J*_sc_ under various light intensities (Figure 3d). *J*_sc_ can be approximated to depend on *I* ^α^, where I represent the light intensity. For α = 1, all charges are being extracted without recombination.^47^ The increased values of α for PEI/MX-based devices indicate reduced recombination when compared to devices using pristine PEI layers (Table 2).

Steady PL and TRPL spectra were also collected to study the charge extraction dynamics of the devices. The introduction of MXenes into the PEI layer progressively quenches PL emission indicating a facilitated extraction of carriers through perovskite/PCBM/PEI/MX interfaces (Figure 3e). This observation is consistent with the faster TRPL decay observed for MXene-containing samples. The TRPL curves (Figure 3f) were fitted by a biexponential decay kinetics (eq S3), and the results are summarized in Table S1. TRPL decay typically involves two characteristic lifetimes: τ_1_ (fast component) and τ_2_ (slow component). The fast component (τ_1_) is commonly associated with interfacial processes, including charge transfer and interfacial recombination, whereas the slow component (τ_2_) is generally attributed to bulk recombination within the perovskite layer. The τ_1_ values show minimal variation for the PEI/MX samples, while an increase in the amplitude of the fast component is observed. This behavior indicates a larger contribution of fast decay pathways, which may be associated with enhanced interfacial charge transfer. However, contributions from interfacial recombination cannot be excluded. In addition, τ_2_ shows a slight increase for PEI/MX samples, which may suggest reduced bulk recombination, possibly related to improved interfacial properties. The average lifetimes (τ_r_, eq S4) are summarized in Table S1, with PEI/MX-1.5% devices exhibiting slightly shorter τ_r_ (3.87 ns) compared to pristine PEI (4.02 ns). These findings are consistent with the shorter charge extraction times (τ_c_) observed for PEI/MX-based devices, as obtained from transient photocurrent (TPC) measurements (Figure S7 and eq S5). Overall, the combined TRPL and TPC results suggest improved charge extraction in PEI/MX-based devices.

Recombination losses were further investigated using the trap-filled-limited voltage (*V*_TFL_) estimated from the double-logarithmic plot of the dark *J*–*V* characteristics of the devices (Figure S8). The trap-densities (*N*_t_) were calculated according to eq S6 and the results are given in Table 2. The incorporation of MXenes into the PEI layer leads to a reduced trap density (*N*_t_), reaching a minimum value of 4.15 × 10^17^ cm^–3^ for PEI/MX-1.5%-based devices, compared to 5.01 × 10^17^ cm^–3^ for devices with pure PEI. This apparent decrease in defect density is consistent with the observed shorter charge-extraction times, indicating that the PEI/MX buffer layer facilitates more efficient charge injection into the electrode. As a result, nonradiative recombination at the interfaces, primarily caused by energy-level misalignment and charge-carrier back transfer, is significantly reduced. Additionally, optimized interfacial improvements between PEI/MX and PCBM could lead to passivation of interfacial defects and therefore recombination centers.^48,49^ As shown by AFM analyses and Raman mapping, we observed no considerable changes in morphological and topographical characteristics between PEI and PEI/MX layers. Given that MXene enhances the hydrophilicity of PEI through its terminal –F, –OH, and –O functional groups, a more uniform dispersion of the buffer layer on PCBM is achieved, facilitated by the polar nature of the IPA solvent.

### 3.3 Charge Transport Properties and Stability

To investigate the influence of PEI/MXene interactions on the electrode work function and charge transport, surface photovoltage (SPV) measurements (Figure S9) were performed, and the respective work function values were calculated using eqs S7 and S8. Under dark conditions, the Ag sample exhibits a work function (WF) of 4.1 eV, which remains unchanged upon illumination. The introduction of PCBM does not significantly affect the metal WF, either initially or under light exposure. However, upon returning to dark conditions, a slight shift toward lower WF values is observed, followed by rapid relaxation. This behavior can be attributed to interfacial charge accumulation arising from the energy level mismatch between Ag and PCBM.^6^

The incorporation of PEI induces a pronounced shift in the apparent WF of the Ag/PEI interface, from 4.1 eV (bare Ag) to 3.85 eV. Upon illumination, WF rapidly decreases, followed by a gradual increase until stabilization at 3.77 eV. When the light is switched off, a transient overshoot in the opposite direction is observed, after which the WF returns to its initial dark-state value. In contrast, the incorporation of MXene does not induce any further shift in the WF under dark conditions. Under illumination, the initial decrease observed in PEI-only samples is preserved; however, the subsequent gradual increase is suppressed. Instead, the WF remains nearly constant during illumination, yielding a steady-state variation of ∼50 meV. Upon switching off the light, the WF rapidly recovers its original dark-state value without any observable overshoot.

It is well-known that PEI introduces an interfacial dipole at the electrode interface, thereby modifying the WF and inducing band bending in the adjacent semiconductor.^22,23^ Our results demonstrate that the incorporation of MXene significantly alters this behavior. In PEI/MXene blended layers, electrons are likely preferentially injected into MXene under device operation due to its higher work function (5.1 eV, as measured by Kelvin probe), allowing it to act as a partial charge reservoir. The presence of this additional electron reservoir within the PEI matrix enhances the local electric field and combined with the high conductivity and strong polarizability of MXene, facilitates rapid charge redistribution while suppressing trap-assisted charge accumulation at the interface. As a result, the system reaches a steady state more rapidly. This mechanism resembles the behavior reported for metallic nanoparticles embedded into semiconductors.^47–49^

The dynamics of the photogenerated carriers were investigated using charge extraction by linearly increasing voltage (photo-CELIV). Charge carriers were generated by a 50 μs light pulse and subsequently extracted from the devices by applying a linearly ramped bias voltage at varying sweep rates. The photogenerated carrier mobility (μ) is then calculated using the following equation^50^

1 $$\mu =\frac{d^{2}}{2A{t_{\max}}^{2}}{\left[\frac{1}{6.2\left(1+0.002\frac{\Delta J}{J_{0}}\right)}+\frac{1}{\left(1+0.12\frac{\Delta J}{J_{0}}\right)}\right]}^{2}$$

here *d* is the PSC thickness, *A* corresponds the applied voltage sweep ramp, *t*_max_ indicates the time at current maxima and Δ*J* refers to the current increment from the dark-CELIV measurement (*J*_0_). Figure 4a shows the current density vs time curves acquired from the photo-CELIV measurements at different voltage ramp rates. Surprisingly, PEI/MX-based PSCs displayed peak maxima at longer times, indicating that a higher voltage is required for most efficient charge extraction compared to pure PEI based devices. These results can be illustrated by plotting the calculated apparent mobilities μ versus the ramp rate (Figure 4b). As example, at a voltage ramp rate of 50 k V s^–1^, PEI/MX-1.5%-based devices show slower μ (4.8 × 10^–4^ cm^2^ V^–1^ s^–1^) as compared to solar cells using pristine PEI layers (8.2 × 10^–4^ cm^2^ V^–1^ s^–1^). Despite the reduced apparent mobility, PEI/MX-based devices exhibit a considerable increase in the extracted charge density (*n*) obtained by integrating the transient photo-CELIV curves (Figure 4c) PEI/MX-1.5%-based solar cells exhibit a significantly higher carrier density (9.2 × 10^15^ cm^–3^) compared to PEI (4.4 × 10^15^ cm^–3^). This increase is consistent with the higher *J*_sc_ values observed in the MXene-based PSCs. (Table 1).

**Figure 4:**
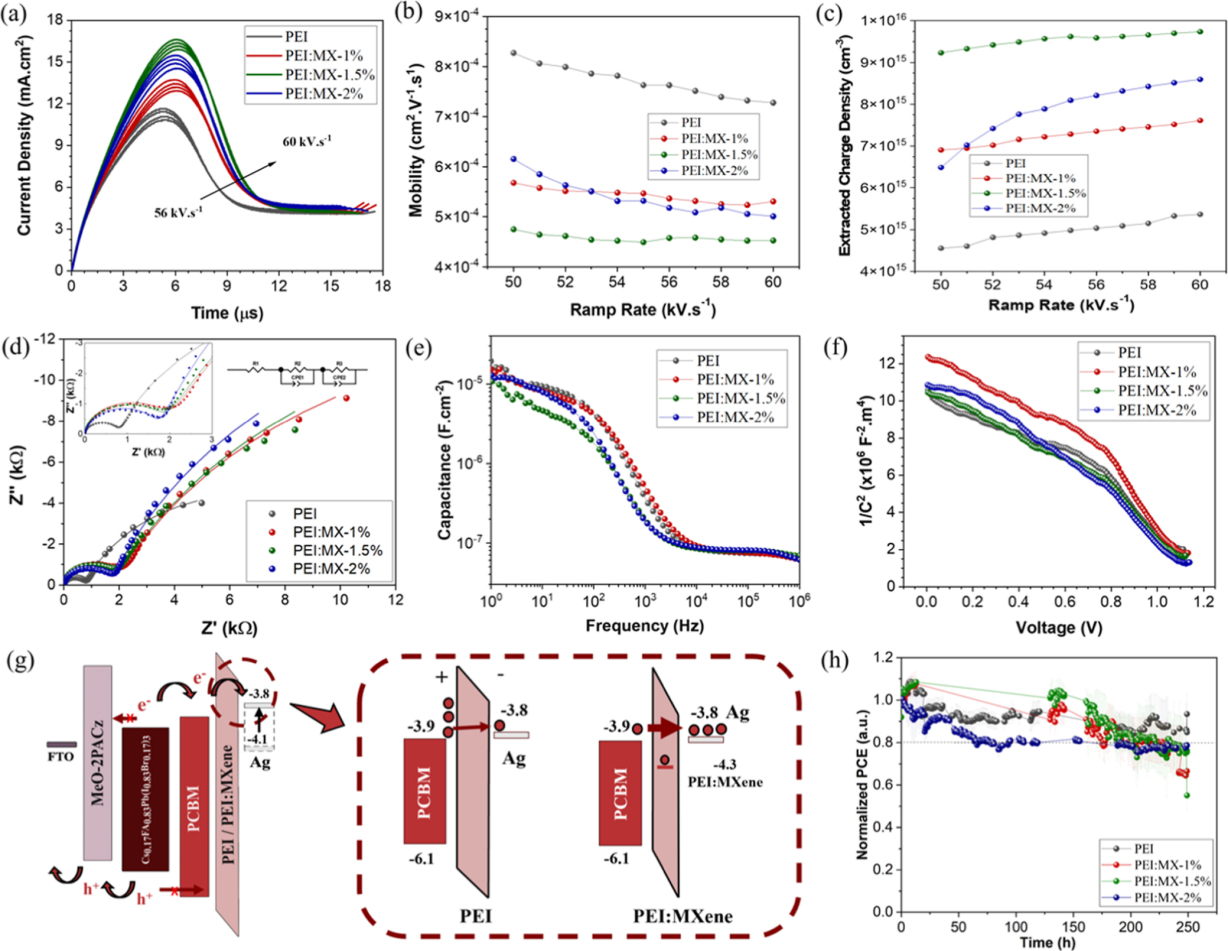
Photo-CELIV: current density vs time curves at varying ramp rates (a), calculated charge mobility (b) and density of extracted charges (c). (d) Nyquist plots of the PSCS under illumination. (e) Capacitance vs frequency curves carried out under illumination. (f) Mott–Schottky curves from the capacitance behavior in the dark. The IS were collected at 0 V bias with AC perturbation of 50 mV. (g) Schematic illustration of energy levels and charge transport for PEI and PEI/MX PSCs. PCBM energy levels were extracted from.^51^ (h) Normalized PCE-time curves for PEI and PEI/MX (1, 1.5 and 2%) obtained by ISOS-L-2 stability tests.

To better understand the reduced mobility (μ) along with an increased *n* for PEI/MX-based PSCs, photo-CELIV curves were measured under different light intensities. Light pulses of 50 μs were applied while maintaining a constant ramp rate of 55 kVs^–1^. Figure S10a shows a general trend observed for all devices, namely that μ decreases substantially as light intensity increases. At low illumination intensity (10%), the extracted mobility of the PEI/MX-1.5% (16.6 × 10^–4^ cm^2^ V^–1^ s ^–1^) device is higher than that of PEI (14.6 × 10^–4^ cm^2^ V^–1^ s ^–1^). As expected (Figure S10), this indicates improved charge transport under low generation conditions. However, with increasing light intensity, a pronounced decrease in apparent mobility is observed, eventually converging to values comparable to those obtained from Photo-CELIV measurements at high voltage ramp rates (55 kVs^–1^). Furthermore, the extracted charge density as a function of light intensity exhibits a nonlinear dependence and tends to saturate at higher illumination levels (Figure S10b).

This trend can be quantitatively correlated with the evolution of the normalized current overshoot, Δ*j*/*j*_0_ (Figure S11), which serves as an indicator of space-charge effects. At 10% illumination, Δ*j*/*j*_0_ < 1, corresponding to a near-ideal regime with minimal field distortion. In contrast, at higher light intensities, Δ*j*/*j*_0_ exceeds unity, marking the transition to a space-charge-dominated regime, in which the internal electric field becomes significantly distorted by accumulated charge. Consistently, the values extracted from Photo-CELIV measurements are Δ*j*/*j*_0_ = 1.8 for PEI, 2.4 for PEI/MX-1%, 3.2 for PEI/MX-1.5%, and 2.6 for PEI/MX-2% (Table S2), clearly indicating strong space-charge conditions across all systems. In this regime, the increasing overshoot leads to enhanced field screening and nonuniform electric field profiles, which are accounted for in the CELIV mobility model through empirical correction factors of the form (1 + *a*·Δ*j*/*j*_0_)^−1^.

The apparent reduction in mobility with increasing light intensity primarily reflects the growing influence of space-charge effects rather than an intrinsic degradation of charge transport properties. The extracted values are consistent with the CELIV analysis discussed above, as well as the SPV findings and TPC charge extraction times.

Trapping states serve as sites for charge recombination, limit charge extraction and, as a result, reduce device performance.^52^ PEI buffer layers are known to reduce the WF of metal electrodes, favoring charge transport and extraction from PCBM to the metal electrode. In addition to morphological observations, the electrical and spectroscopic analyses indicate that the introduction of MXene passivates interfacial defects within the devices. The combination of these merits reduces recombination losses, resulting in longer charge carrier lifetimes and enhanced photogenerated charge extraction. Impedance spectroscopy offers further insights into the electrical properties of the devices. The spectra were acquired under illumination at 0 V and are presented as Nyquist plots in Figure 4d. The equivalent circuit depicted in the inset was used to determine the charge transport (*R*_ct_) and recombination (*R*_rec_) resistances. All devices exhibit *R*_ct_ values below 2 kΩ, indicating that charge transport across the interfaces remains generally efficient regardless of MXene content. *R*_ct_ increases upon MXene incorporation compared to the control but shows a decreasing tendency as the MXene concentration rises, indicating that the effect on charge transport is not monotonic. In contrast, *R*_ct_ exhibits a sharp increase from 12.22 kΩ (PEI) to 34.43 kΩ (PEI/MX-1%), while further increases in MXene content led to only minor changes, reaching 37.55 kΩ and 37.74 kΩ for 1.5% and 2%, respectively (Table S2). This progressive enhancement of recombination resistance implies a substantial suppression of nonradiative carrier recombination, which likely contributes to improved photovoltaic performance consistent with the other characterization measurements.

Capacitance–frequency (*C*-–*f*) measurements under illumination are provided in Figure 4e as additional evidence for the reduction of trap states across the devices. At high frequencies, a plateau corresponding to the intrinsic dielectric response of the bulk perovskite (*C*_bulk_)^53^ is observed, consistent with the expected polarization effect of the perovskite semiconductor. In contrast, the low-frequency region reflects slower interfacial processes involving ionic and electronic transport dynamics.^54^ The similar values of *C*_bulk_ across all devices indicate that the bulk perovskite composition and quality remain similar. However, at low frequencies, the PEI/MX-1.5%-based device exhibits a noticeable reduction in capacitance, suggesting diminished interfacial charge accumulation. This response could be attributed to enhanced charge extraction and reduced trapping at the PCBM/PEI/Ag interfaces likely facilitated by the optimized MXene incorporation.

Mott–Schottky analysis was performed to further investigate the electrostatic modifications induced by the incorporation of MXenes into the PEI buffer layer. To ensure reliable extraction of the depletion capacitance, capacitance–voltage (*C*–*V*) measurements were conducted in the dark at frequencies within the dielectric response range (10 kHz, 31.6 kHz, and 100 kHz), as shown in Figure S12. Among these, 100 kHz was selected for subsequent analysis to minimize contributions from low-frequency interfacial polarization and ionic effects. The built-in potential (*V*_bi_) was extracted from the linear region of the *C* ^2^ vs *V* plot (Figure 4f), revealing an increase from 1.16 V for PEI-based devices to 1.20 V for PEI/MX-1.5% devices (Table S2).

Changes in the electrostatic environment upon MXene incorporation are consistently evidenced by both Mott–Schottky and SVP measurements. SVP analysis (Figure S9) shows a surface potential shift of approximately 50 meV, indicative of an interfacial dipole associated with charge localization near the surface. In contrast, Mott–Schottky analysis probes the buried junction and reveals an increase in *V*_bi_ of approximately 40 meV. To quantitatively estimate the associated charge density, eqs S9,S10 and S12 were employed. Based on Gauss’s law, the observed 50 meV surface potential shift corresponds to an interfacial charge density of ∼1.06 × 10^12^ e·cm^–2^. Independently, *C*–*V* measurements (Figure S12b) were used to estimate the charge density under open-circuit conditions via the capacitor relation (eq S12), yielding a difference of ∼1.5 × 10^11^ e·cm^–2^ between PEI/MX-1.5% and PEI devices. Although this value is approximately 1 order of magnitude lower than the SPV estimation, both approaches are in reasonable agreement and consistently support the observed increase in *V*_bi_, supporting charge storage on MXene. These electrostatic modifications are further supported by CELIV measurements, where the increased interfacial charge density contributes to electric field redistribution and screening effects, thereby influencing the extracted mobility values. Finally, while *V*_bi_ and *V*_oc_ are generally correlated, the latter is typically lower in practical devices due to recombination losses, trap states, and nonideal contacts. In this context, electrical and impedance analyses indicate reduced recombination and decreased trap-assisted losses in MXene-containing devices, which is consistent with the observed increase in *V*_oc_ from 1.10 V (PEI) to 1.12 V (PEI/MX-1.5%).

The improvements in electronic properties discussed above are visually summarized in Figure 4g, which integrates key electrical and interfacial insights across devices without and with MXene. As shown in Figure 4g, the introduction of MXene into the PEI buffer layer creates favorable interfacial states by better accepting charges, that ultimally facilitate electron tunneling across the PCBM/PEI/Ag interfaces. These interfacial improvements are corroborated by device-level photovoltaic metrics: an increase in open-circuit voltage (*V*_oc_), a significant enhancement in fill factor (FF), and an improved *J*_sc_ observed by *J* × *V* curves and EQE analyses which are consistent with reduced recombination losses. Furthermore, photo-CELIV measurements reveal that the PEI/MX-1.5%-based device supports the highest extraction density of photogenerated carriers. Complementary biexponential fitting of TRPL decays confirms faster charge carrier extraction in the same device. Together, these findings reinforce the hypothesis that MXene incorporation in the passivation layer not only suppresses recombination but also improves carrier extraction dynamics, ultimately leading to enhanced device performance.

The density of defects (*N*_tls_) provides further insight into the reduction of trap states in the MXene-enhanced PSCs. *C*–*f* measurements under dark conditions, shown in Figure S10a, were used to extract the density of states (DOS) spectra, presented in Figure S10b. These results reveal a significant decrease in *N*_tls_ upon incorporation of MXene into the PEI buffer layer. In particular, the PEI/MX-1.5%-based device exhibits a lower trap density of 3.07 × 10^17^ eV^–1^ cm^–3^, compared to 9.45 × 10^17^ eV^–1^ cm^–3^ for the pristine PEI-based control. The significant reduction in trap-state density for this composition is consistent with the lowest trap-filled limit voltage obtained from space-charge-limited measurements.

Surprisingly, the improvement in PCE performance was not reflected in more stable devices. Encapsulated PEI/MX-based PSCs exhibited similar stability to devices using pristine PEI under continuous light irradiation at 65 °C (ISOS-L2). As shown in Figure 4h, both PEI and PEI/MX-1.5% devices withstood about 250 h before reaching T80. Moreover, a higher MXene concentration (PEI/MX-2%) led to less stable devices. These results were unexpected, since the reduction of recombination sites at PCBM/PEI/MX/Ag would mitigate trapped charge induced degradation at the interface.^55^ ETL and buffer layers such as PCBM and BCP generally do not fully block ion migration, allowing halide ions to eventually reach the metal electrode.^34^ These ions can react with the silver electrode to form insulating silver halides, increasing the contact resistance and progressively corroding the electrode, thereby compromising device performance and stability. Similarly, PEI, being a cationic polyelectrolyte, may facilitate halide migration and further contribute to electrode oxidation.^56^

On the other hand, MXenes may progressively oxidize through interactions with oxygen and moisture. The formation of MXene-derived TiO_2_ domains can potentially participate in photocatalytic or redox reactions that generate reactive species capable of degrading organic molecules or accelerating metal corrosion.^57^ Nevertheless, when MXene is embedded within the PEI polymer matrix and further protected inside the PSC architecture, degradation associated with MXene oxidation is expected to occur on longer time scales, as reported in previous studies.^29,34,35^ From a device performance perspective, these processes can introduce additional trap states and interfacial degradation, hindering charge transport and extraction, which is reflected in the reduction of *J*_sc_ (Figure S14). Simultaneously, the deterioration of interfacial contact and the increase in resistive losses contribute to a decline in the FF (Figure S14). Additionally, the formation of defect states may enhance nonradiative recombination pathways, leading to a gradual decrease in *V*_oc_ (Figure S14), although this effect is less pronounced compared to the variations observed in *J*_sc_ and FF.

Therefore, considering the similar stability trends observed for all devices, the relatively limited stability of the cells is more likely related to the intrinsic characteristics of the PEI interlayer. While the incorporation of MXene improves the electrical performance of PEI/MX-based devices, it is noteworthy that the *V*_oc_ remains comparatively more stable over time and exhibits consistently higher values in MXene-containing devices. This behavior is consistent with reduced nonradiative recombination losses, as supported by our experimental findings, although it does not fully suppress the overall degradation mechanisms. Nevertheless, additional strategies will be required to enhance their operational stability. Future work should explore complementary buffer or protective layers that can effectively suppress ion migration and improve long-term durability of this system.

## 4 Conclusions

High performance inverted PSCs were fabricated by introducing PEI/MX cathode buffer layers. Structural and spectroscopic analyses of PEI/MX films confirmed a favored interaction between the 2D material and PEI. The PEI/MX-based PSC exhibited enhanced photovoltaic parameters, inferring better extraction of charges and suppression of recombination losses. These effects contributed to enhancing the PCE of the devices to 22.0 ± 0.8% (23.1% for the best PSC). The PEI/MX buffer layer provides better interfacial features and favorable energy alignment with PCBM and the metal electrode. In addition, the charge trapping by MXenes acts by preventing recombination losses at PCBM/PEI/MX/Ag interfaces. The suppression of recombination losses results in a longer charge carrier lifetime. The enhanced extraction of charges was confirmed by the transient currents of the photogenerated charges from photo-CELIV, in addition to the rapid decay curves of TPC and TRPL analyses. The elevated recombination resistance (*R*_rec_) extracted from EIS substantiates the effective suppression of carrier recombination in PEI/MX. In addition, DOS analysis confirms a reduction in trap-state density for PEI/MX. Despite improving PCE performance, PEI/MX buffer layers did not lead to enhanced stability under the ISOS-L2 protocol. Nevertheless, the results obtained in this work demonstrate that solution-processable PEI/MX is a promising material for efficiently replacing conventional cathode buffer layers such as BCP in *p*–*i*–*n* PSCs. Future studies should focus on strategies to enhance the stability of PEI-based devices.

## Supplementary Material



## Notes

Figure S1: XDR patterns of Ti_3_AlC_2_ MAX phase and Ti_3_C_2_T_*x*_ MXene. Figure S2: FE-SEM images of Ti_3_C_2_T_*x*_ MXene flakes using In-Lens detector at magnifications of (a) 2k×, (b) 15k×, (c) 35k× and (d) secondary electron detector (SE) at 35k×. Figure S3: Contact angle (CA) images of fully integrated PSC with PEI and PEI/MX (1%, 1.5% and 2%) buffer layers to water. Figure S4: Box plots of photovoltaic parameters (a) *J*_SC_ (mA cm^–2^), (b) *V*_OC_ (V), (c) FF (%) and (d) PCE (%) of PSCs employing PCBM and PCBM/PEI (10, 5, 2, and 0.2 mg mL^–1^). Figure S5: Box plots of photovoltaic parameters (a) *J*_SC_ (mA cm^–2^), (b) *V*_OC_ (V), (c) FF (%) and (d) PEC (%) of at least seven PSCs for each configuration. Figure S6: *I*–*V* and differential resistance versus voltage curves of (a) PEI and (b–d) PEI/MX (1, 1.5 and 2%) based PSCs, respectively. Figure S7: TPC curves of PEI and PEI/MX (1, 1.5 and 2%) based devices. Table S1: Fitted parameters of the TRPL of perovskite/PCBM/PEI and perovskite/PCBM/PEI/MX (1, 1.5 and 2%) films. Fitted extraction charges times (*t*_c_) of TPC curves of PSCs under simulated irradiation (72 mW cm^–2^). Figure S8: Dark log–log *I*–*V* curves of (a) PEI and (b–d) PEI/MX­(1, 1.5 and 2%), showing respective the trap-filled limited potential (*V*_TFL_) and trap density (*N*_t_), respectively. Figure S9: Transient photoinduced surface potential (SPV) from the Ag surface potential measured with PCBM, PEI, and PEI-MXene samples. Light on and Light Off legends indicate the time when the light shutter was open and closed, respectively. 50 meV represents the difference in work function of the samples containing PEI/MX-1.5% and pure PEI. Figure S10: (a) Mobility and (b) extracted charge density obtained from light intensity–CELIV at 55 kV/s ramp of PEI and PEI/MX PSCs, respectively. Figure S11: Overshoot current number from different light intensities (10–100%) CELIV at 55 kV/s ramp of (a) PEI, (b) PEI/MX-1%, (c) PEI/MX-1.5% and (d) PEI/MX-2% PSCs. The dashed line corresponds to the threshold where space charge dominates mobility values over time max. Table S2: Device’s overshoot current number (Δ*j*/*j*_0_) from photo-CELIV at 55 kV/s ramp, charge-transfer resistance (*R*_ct_), recombination resistance (*R*_rec_), number of charges calculated from *C*–*V* (*N*_*C*_–_*V*_) at *V*_OC_, built-in potential (*V*_bi_ ), capacitance at 1 HZ values (C_1 Hz_) and density-of-stated (DOS) of PEI and PEI/MX based PSCs. Figure S12: (a) Capacitance–voltage curves of PEI PSC at 10, 31.6, and 100 kHz. (b) Capacitance–voltage curves of PEI and PEI/MX (1, 1.5 and 2%) devices at 100 kHz. The measurements were carried out in the dark at room temperature and under 50 mV of AC stimulus. Figure S13: (a) *C*–*f* spectra and (b) density-of-states (DOS) of the PEI and PEI/MX­(1, 1.5 and 2%) PSCs. The spectra were measured at room temperature, under dark conditions and at 0 V bias, with AC signal of 50 mV. Figure S14: Normalized *J*sc, *V*oc and FF-time curves for PEI and PEI/MX (1, 1.5 and 2%) obtained by ISOS-L-2 stability tests (PDF)
