# Supplementary material for: Charge-Transfer Enhancement by PEI/MXene Buffer Layer for Boosting Performance of Inverted Perovskite Solar Cells
Source: ACS Appl Mater Interfaces. 2026 May 12;18(20):28638–50. doi: 10.1021/acsami.6c01017 (PMC13220228; doi:10.1021/acsami.6c01017)
Supplement: Supplementary file 1 [file am6c01017_si_001.pdf]

Supporting Information

Charge-Transfer Enhancement by PEI/MXene Buffer Layer for Boosting Performance of Inverted Perovskite Solar Cells

João P. F. Assunção[a,b,c], Hugo G. Lemos[a,d], Jessica H. H. Rossato[a,], Gabriel L. Nogueira[a], Mirjana Dimitrievska[e], Marcos A. Cruz Jr.[a], Silvia L. Fernandes[f], Sidney A. Lourenço[g], Frank Nüesch[b,c], and Carlos F. O. Graeff[a*]*

[a] São Paulo State University (UNESP), School of Sciences, Department of Physics, Bauru, SP, 17033-360, Brazil

[b] Empa – Swiss Federal Laboratories for Materials Science and Technology, Laboratory of Functional Polymers, Dübendorf 8600, Switzerland

[c] Institute of Materials Science and Engineering, Ecole Polytechnique Fédérale de Lausanne (EPFL), Station 12, CH-1015 Lausanne, Switzerland

[d] Instituto Tecnológico de Aeronáutica (ITA), Departamento de Geração e Armazenamento de Energia, Fortaleza, 60415-513, Brazil

[e] Empa – Swiss Federal Laboratories for Materials Science and Technology, Nanomaterials Spectroscopy and Imaging Group, Transport at Nanoscale Interfaces Laboratory, Überlandstrasse 129, 8600 Dübendorf, Switzerland

[f] Oninn - Innovation Center, Belo Horizonte, MG, 31035-536, Brazil

[g] Materials Science and Engineering Program (PPGCEM), Federal Technological University of Paraná (UTFPR), Londrina, PR, Brazil

* *Corresponding authors:* hugo.lemos@unesp.br; carlos.graeff@unesp.br

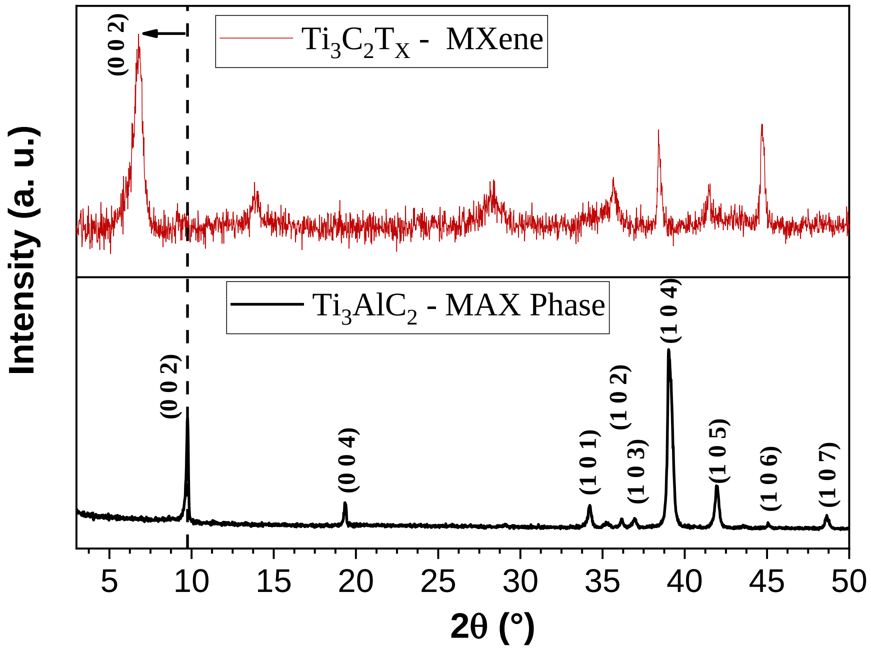

Figure S1: XDR patterns of Ti_3AlC_2 MAX phase and $Ti_3C_2T_x$ MXene.

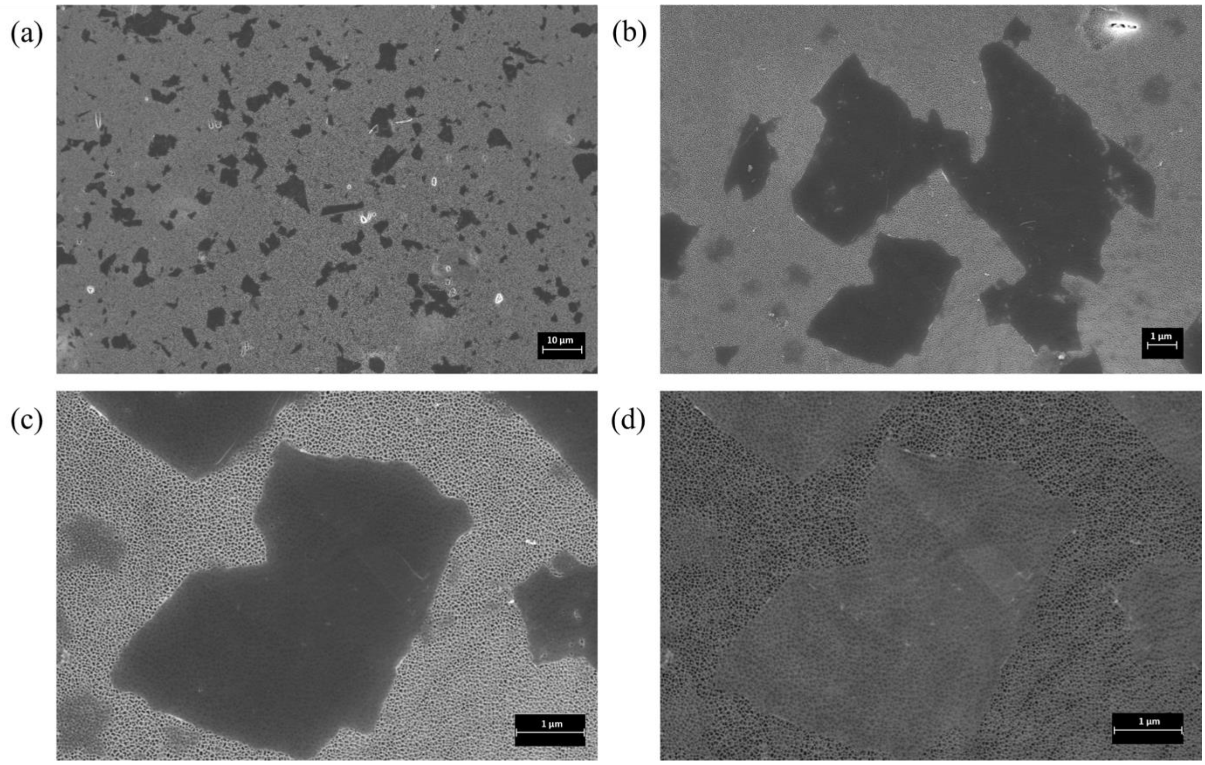

Figure S2: FE-SEM images of $Ti_3C_2T_x$ MXene flakes using In-Lens detector at magnifications of (a) 2k x, (b) 15k x, (c) 35k x and (d) secondary electron detector (SE) at 35k x.

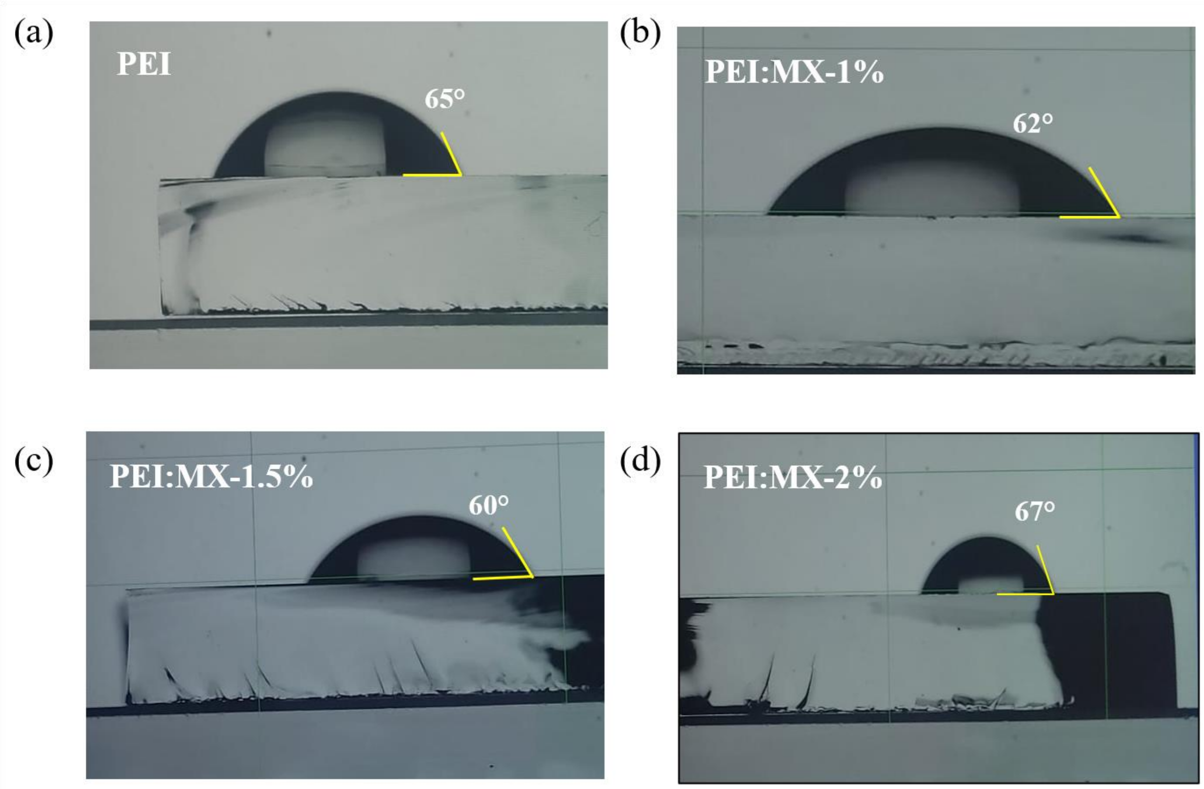

Figure S3: Contact angle (CA) images of fully integrated PSC with PEI and PEI/MX (1%, 1.5% and 2%) buffer layers to water.

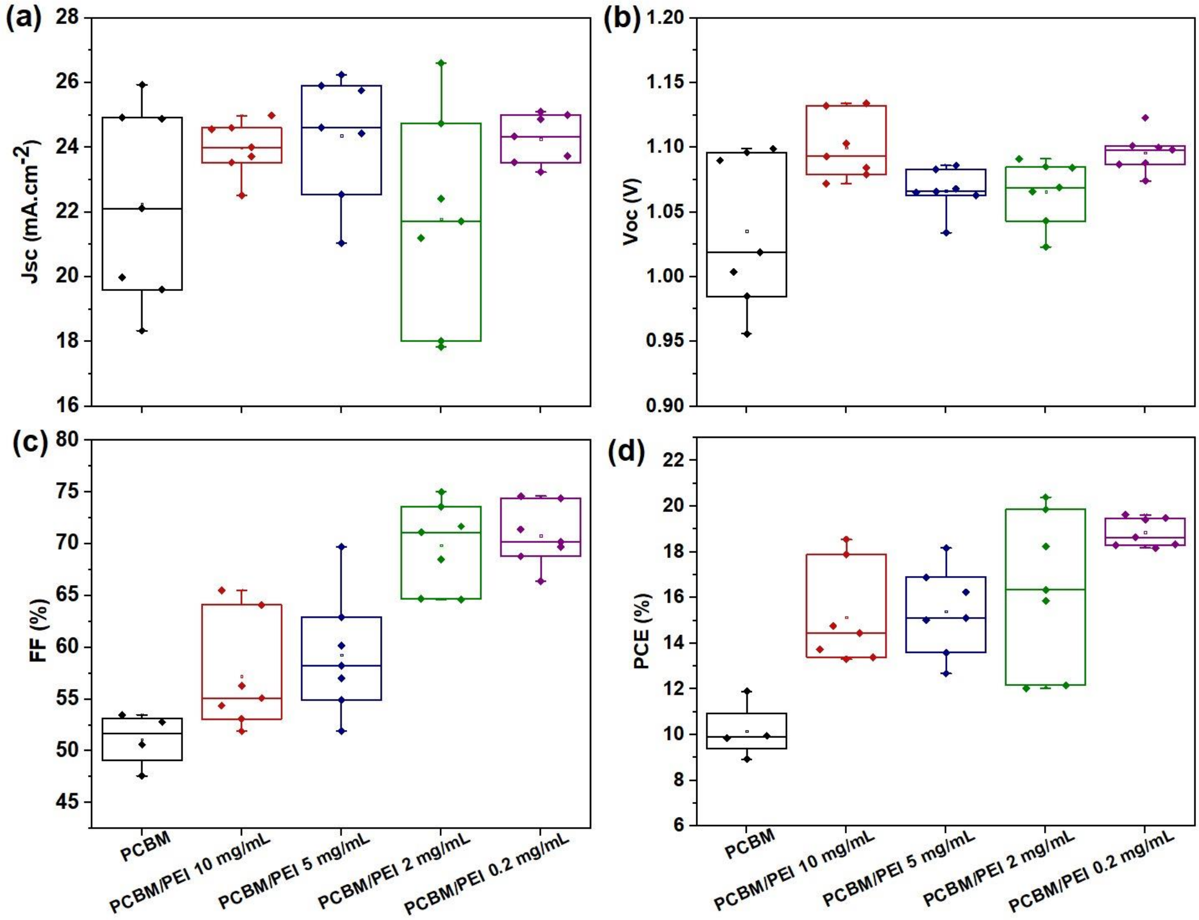

Figure S4: Box plots of photovoltaic parameters (a) J_{SC} (mA.cm^{-2}), (b) V_{OC} (V), (c) FF (%) and (d) PCE (%) of PSCs employing PCBM and PCBM/PEI (10, 5, 2, and 0.2 mg.mL^{-1}).

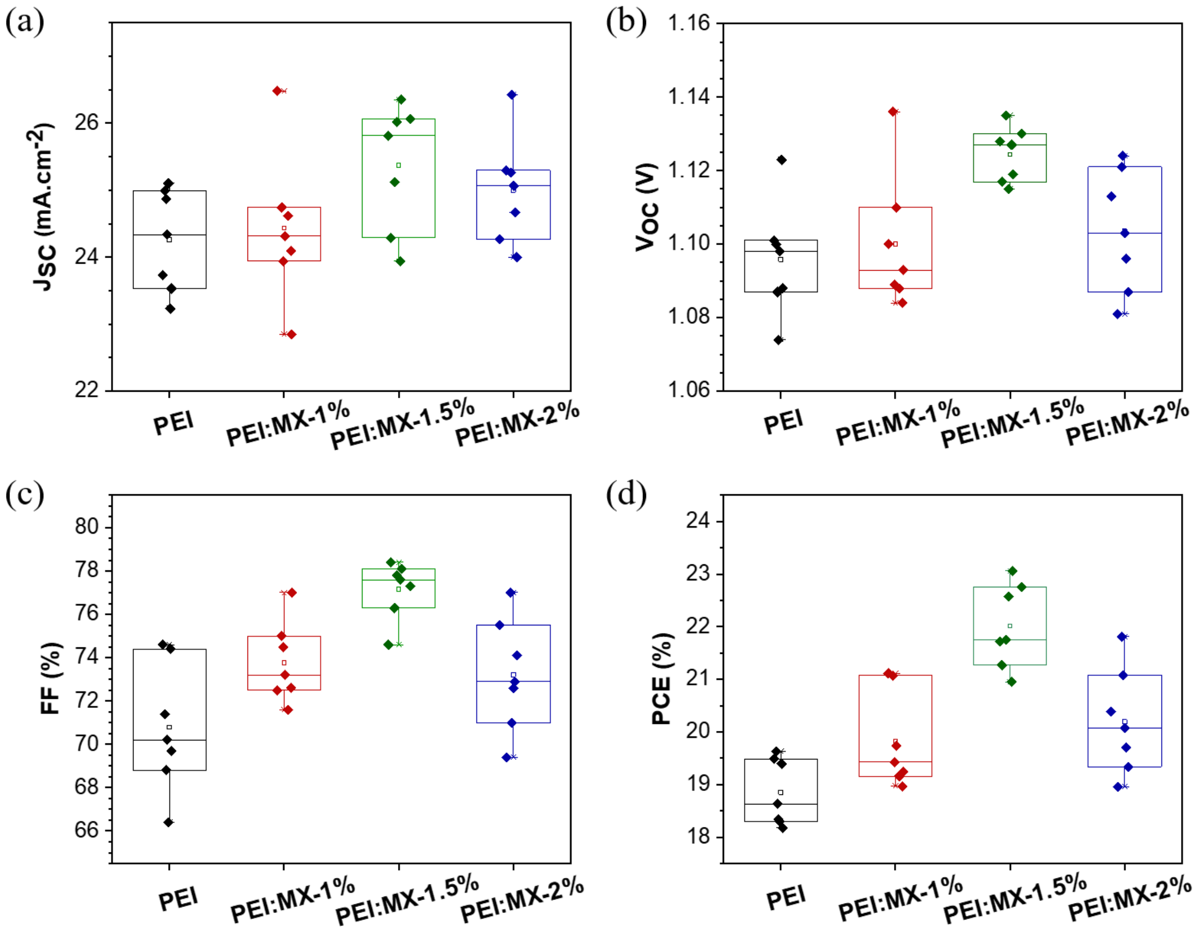

Figure S5: Box plots of photovoltaic parameters (a) J_{SC} (mA.cm^{-2}), (b) V_{OC} (V), (c) FF (%) and (d) PCE (%) of at least seven PSCs for each configuration.

The hysteresis index (HI) calculations were carried out using the following equation:

$$|HI| = \frac{(PCE_{rev} - PCE_{fwd})}{PCE_{rev}} \qquad \text{(Eq. S1)}$$

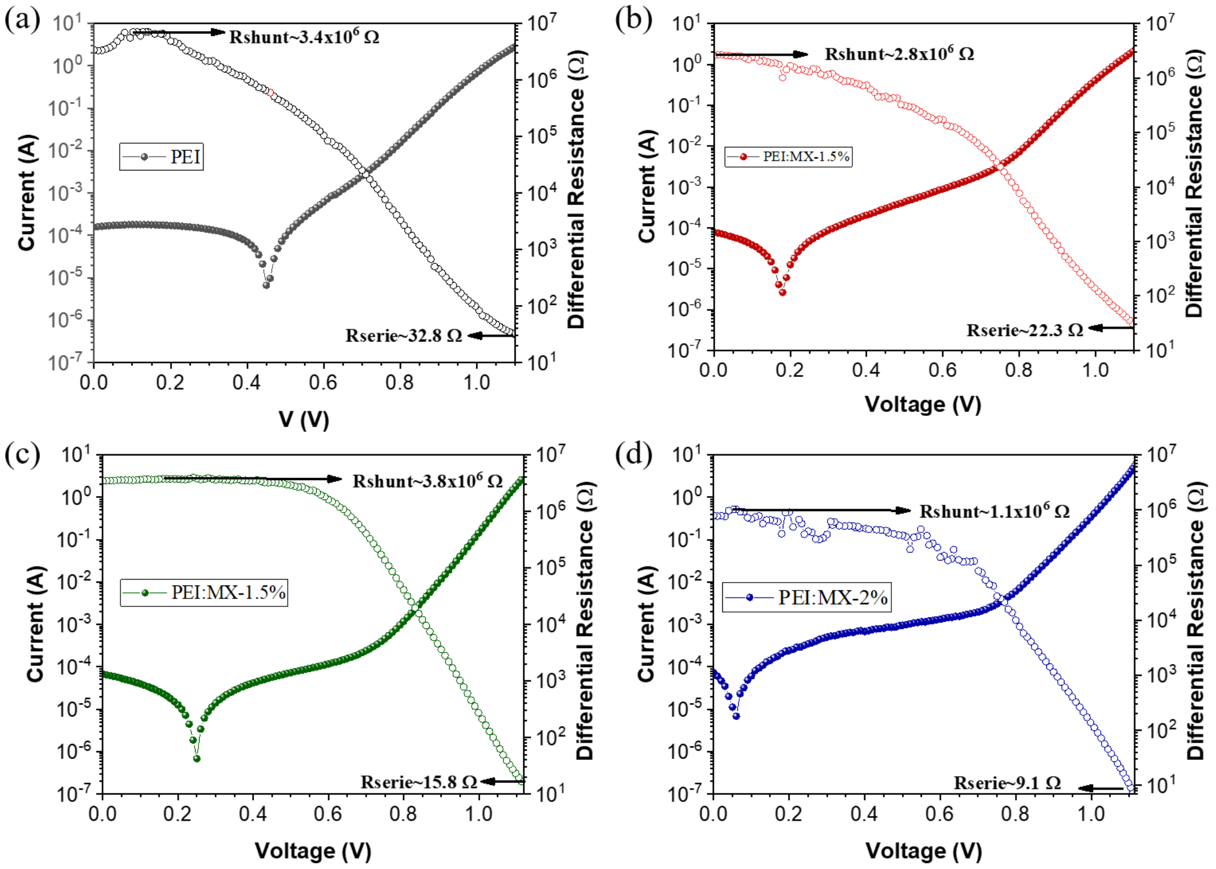

Figure S6: I-V and differential resistance versus voltage curves of (a) PEI and (b-d) PEI/MX (1, 1.5 and 2%) based PSCs, respectively.

$$V_{oc} \propto \frac{nkT}{q} \ln I^2 \qquad \text{(Eq. S2)}$$

Where n is the ideality factor, k is the Boltzmann constant, T corresponds to the device temperature, q is the elementary charge, and I refer to the light intensity.

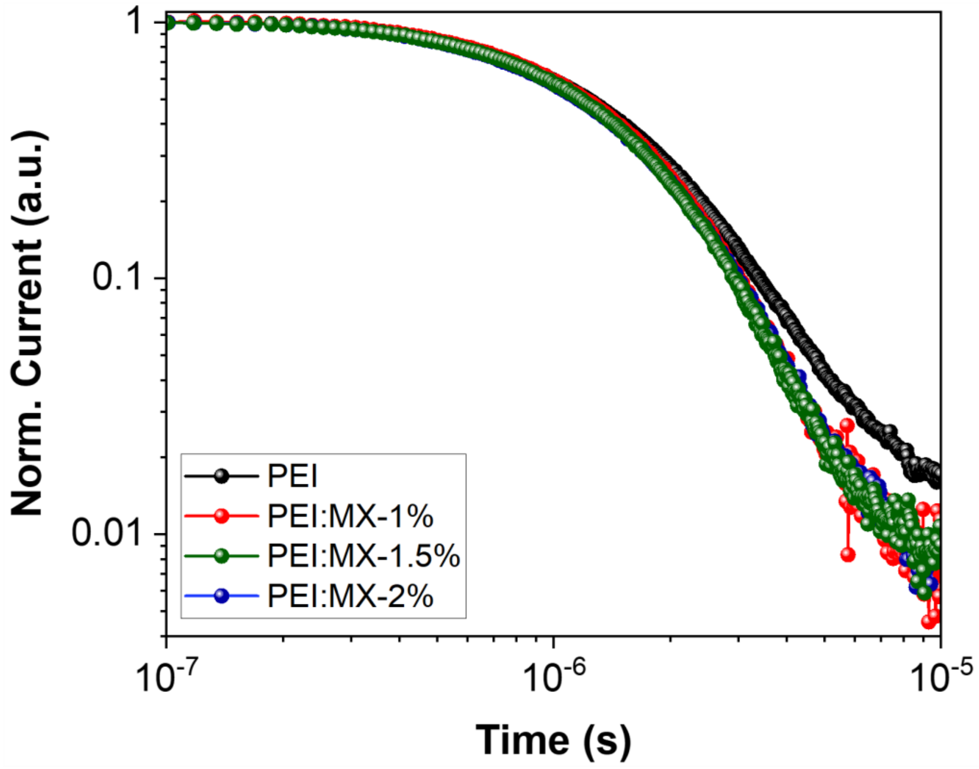

Figure S7: TPC curves of PEI and PEI/MX (1, 1.5 and 2%) based devices.

The TRPL decays were fitted using the following equation,

$$X(t) = A_1 \cdot exp\left(-\frac{t}{\tau_1}\right) + A_2 \cdot exp\left(-\frac{t}{\tau_2}\right)$$

(Eq. S3)

where A_1 and A_2 are constant, t is intensity decay time, τ_1 and τ_2 are the fitted lifetimes.

The average lifetime, τ_x was calculated using the following equation:

$$\tau_r = \frac{A_1{\tau_1}^2 + A_2{\tau_2}^2}{A_1\tau_1 \cdot A_2\tau_2}$$

(Eq. S4)

The current decay was fitted using the following equation,

$$I(x) = A_1 \cdot exp\left(-\frac{x}{t_e}\right)$$

(Eq. S5)

where A_1 is constant, x is I decay time and $\mathbf{t_e}$ is the fitted charge extraction time.

Table S1: Fitted parameters of the TRPL of perovskite/PCBM/PEI and perovskite/PCBM/PEI/MX (1, 1.5 and 2%) films. Fitted extraction charges times (t_c) of TPC curves of PSCs under simulated irradiation (72 mW.cm^{-2}).

Sample	A_1	τ_1 (ns)	A_2	τ_2 (ns)	τ_r (ns)	t_e (µs)
PEI	85.38	0.31	14.62	5.29	4.02	1.36
PEI:MX-1%	91.49	0.35	8.51	6.05	3.85	1.20
PEI:MX-1.5%	91.27	0.35	8.73	6.01	3.87	1.12
PEI:MX-2%	92.89	0.38	7.11	5.99	3.44	1.12

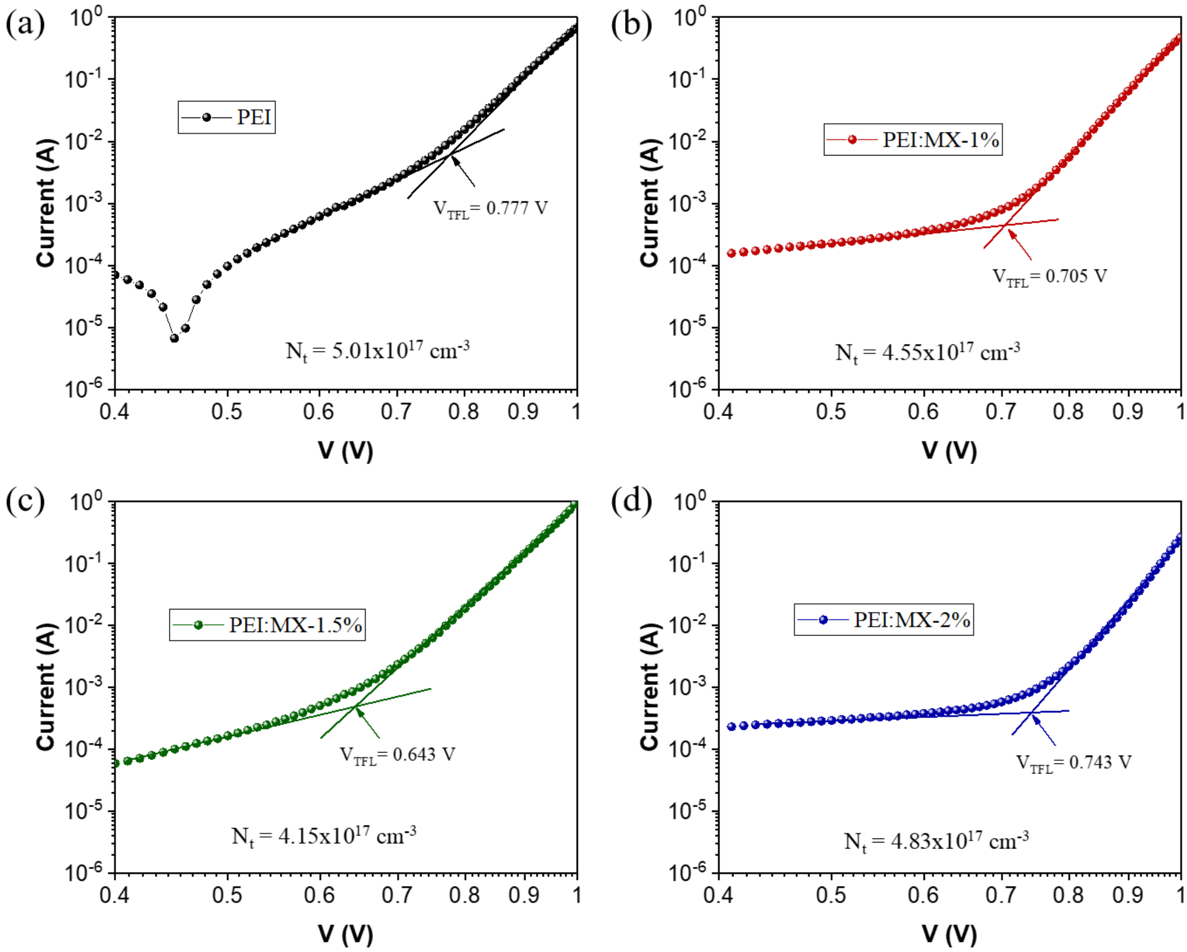

Figure S8: Dark log-log I-V curves of (a) PEI and (b-d) PEI:MX(1, 1.5 and 2%), showing respective the trap-filled limited potential (V_{TFL}) and trap density (N_t), respectively.

$$N_t = \frac{2\epsilon_r\epsilon_0 V_{TFL}}{e\, d^2} \ln \quad I^2 \qquad \text{(Eq. S6)}$$

Where e is the electron charge, d refers to the device thickness, ε_0 is the vacuum permittivity and ε is the dielectric constant of the perovskite (here, set as 21[1]).

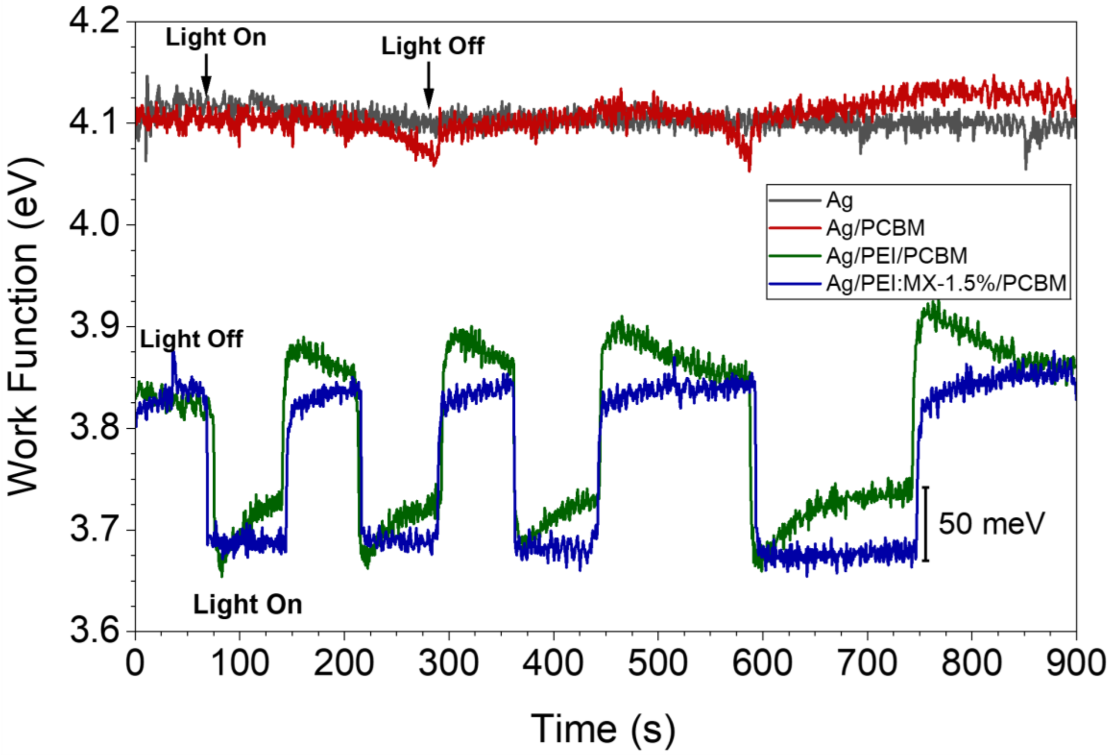

Figure S9: SPV of the Ag surface potential measured with PCBM, PEI, and PEI/MXene samples. “Light on” and “light off” labels indicate the periods during which the light shutter was open and closed, respectively. The 50 meV value represents the difference in work function between PEI/MX-1.5% and pure PEI samples.

The work function (WF) was determined by measuring the contact potential difference (CPD) between the kelvin probe tip (WF_{tip}) and a known WF_{Au} reference (Au = 5.1 eV). Later, the investigated thin film (WF_{Samp}). The CPD is measured with a resolution of 1 mV. Since the WF of the reference is known, the work function of the sample can be calculated as:

$$WF_{tip} = WF_{Au} + e \cdot CPD \qquad \text{(Eq. S7)}$$

$$WF_{samp} = WF_{tip} - e \cdot CPD \qquad \text{(Eq. S8)}$$

The number of charges responsible for the work function shift was calculated from Gauss law:

$$\sigma = \frac{\Delta V \epsilon_0 \epsilon_r}{d} \quad \text{(Eq. S9)}$$

$$\sigma = Ne \quad \text{(Eq. S10)}$$

where d is the thickness of PEI, ΔV is the energy variation, ε_0 is the vacuum permittivity, ε_r is the relative permittivity of the material, e is the electron elementary charge and N is the number of charges.

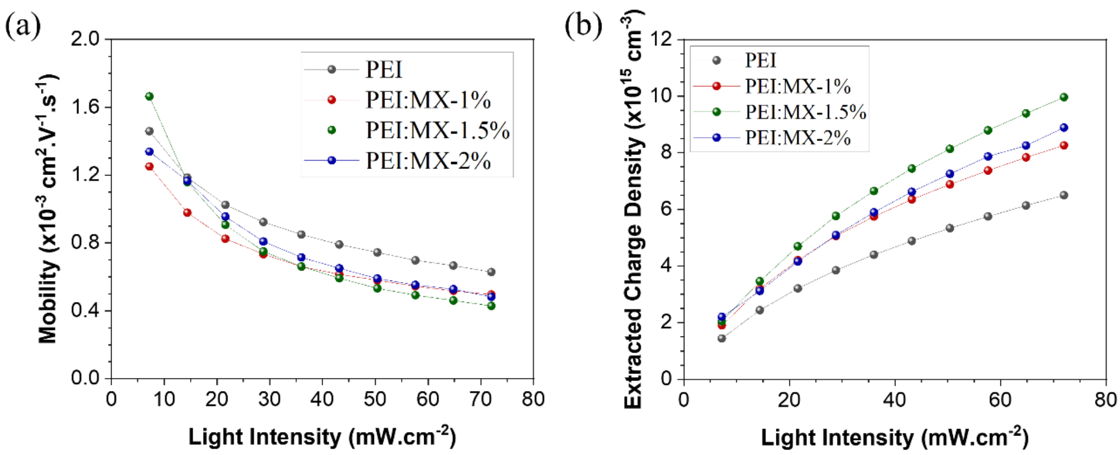

Figure S10: (a) Mobility and (b) extracted charge density obtained from light intensity – CELIV at 55 kV/s ramp of PEI and PEI/MX PSCs, respectively.

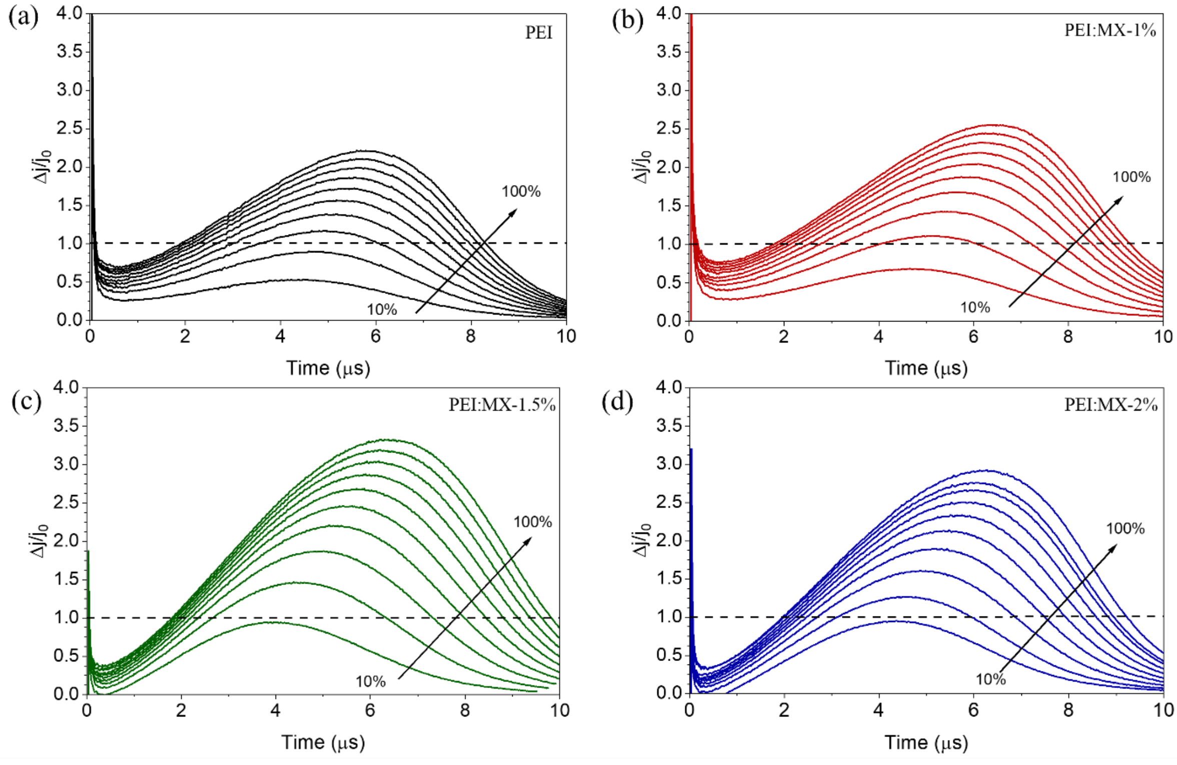

Figure S11: Overshoot current number from different light intensities (10 –100%) CELIV of (a) PEI, (b) PEI/MX-1%, (c) PEI/MX-1.5% and (d) PEI/MX-2% PSCs. The dashed line corresponds to the threshold where space charges dominate mobility values over time max.

Table S2: Device's overshoot current number ($\Delta j/j_0$) from photo-CELIV at 55 kV/s ramp, charge-transfer resistance (R_{ct}), recombination resistance (R_{rec}), number of charges calculated from C-V ($N_{C\text{-}V}$) at V_{OC}, built-in potential (V_{bi}), capacitance at 1 HZ values (C_{1Hz}) and density-of-stated (DOS) of PEI and PEI/MX based PSCs.

Device	$\Delta j/j_0$	R_{ct} (kΩ)	R_{rec} (kΩ)	V_{bi} (V)	$N_{C\text{-}V}$ (cm^{-2})	C_{1Hz} ($F.cm^{-2}$)	DOS ($eV^{-1}.cm^{-3}$)
PEI	1.8	0.77	12.22	1.16	$1.27x10^{12}$	$1.94x10^{-5}$	$9.45x10^{17}$
PEI:MX-1%	2.4	1.84	34.43	1.19	$1.35x10^{12}$	$1.54x10^{-5}$	$5.79x10^{17}$
PEI:MX-1.5%	3.2	1.74	37.55	1.20	$1.42x10^{12}$	$1.04x10^{-5}$	$3.07x10^{17}$
PEI:MX-2%	2.6	1.66	37.74	1.18	$1.43x10^{12}$	$1.29x10^{-5}$	$7.15x10^{17}$

To perform the Mott-Schottky analysis, we obtained C-V curves under 10, 31, and 100 kHz. We focused our analysis on the 100 kHz to overestimate the dielectric constant.

• ε_0 = 8.85 x 10^{-12} F.m^{-1}

• d = 600 nm (PVK thickness)

• C_g = 3.15 x 10^{-4} F.m^{-2} (from the PEI plot in Figure S9-a)

• ε = 21

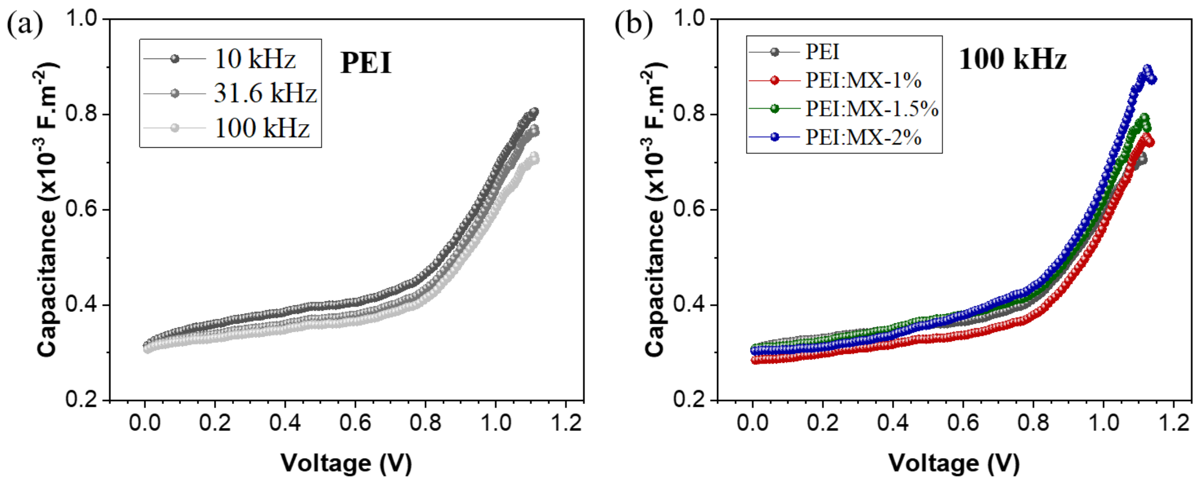

Figure S12: (a) Capacitance-voltage curves of PEI PSC at 10, 31.6 and 100 kHz. (b) Capacitance-voltage curves of PEI and PEI:MX (1, 1.5 and 2%) devices at 100 kHz. The measurements were carried out in the dark at room temperature and under 50 mV of AC stimulus.

The built-in potential (V_{bi}) was calculate using the following equation[2]:

$$C_{dl}^{-2} = \frac{2}{q\varepsilon\varepsilon_0 N}(V_{bi} - V)$$

(Eq. S11)

where C_{dl} is the depletion layer capacitance and N is the charge density. The V_{bi} was determined from the linear region of the C^{-2} vs V plot in Figure 4-f.

The charges per unit area (N_{C-V}) were calculated from C-V curves at V_{OC} potential for each curve using the capacitor equation:

$$N_{C-V} = e\ C\ V\ A \qquad \text{(Eq. S12)}$$

where e is the elementary charge, C is the capacitance per area, V is the voltage and A is the device Area.

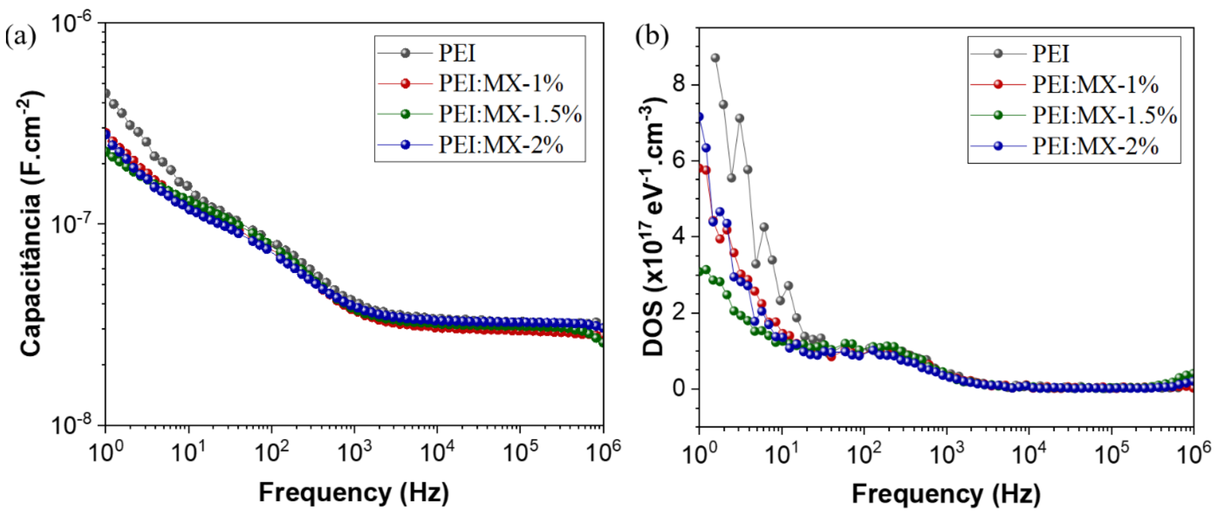

Figure S13: (a) C-f spectra and (b) density-of-states (DOS) of the PEI and PEI:MX(1, 1.5 and 2%) PSCs. The spectra were measured at room temperature, under dark conditions and at 0 V bias, with AC signal of 50 mV.

To calculate the density-of-states (DOS) spectra (Fig. S9) from Eq. S7[3], we took the derivative of the capacitance spectra (Fig. S9-a) and used the V_{bi} values extracted from Fig. 4-f.

$$N_{t,IS} = -\frac{V_{bi}\omega}{qLk_bT}\frac{dC(\omega)}{d\omega} \quad \text{(Eq. S13)}$$

The relation between the demarcation energy (E_ω) and the angular frequency of the applied AC signal (ω) is given by the following:

$$E_\omega = k_bT ln\left(\frac{\omega_0}{\omega}\right) \quad \text{(Eq. S14)}$$

Where ω_0 is a temperature-independent coefficient termed the "attempt to escape frequency". The values of the DOS taken from the low frequency are shown in Table S4. According to Eq. S8, the quantized values are related to deep-level defects.

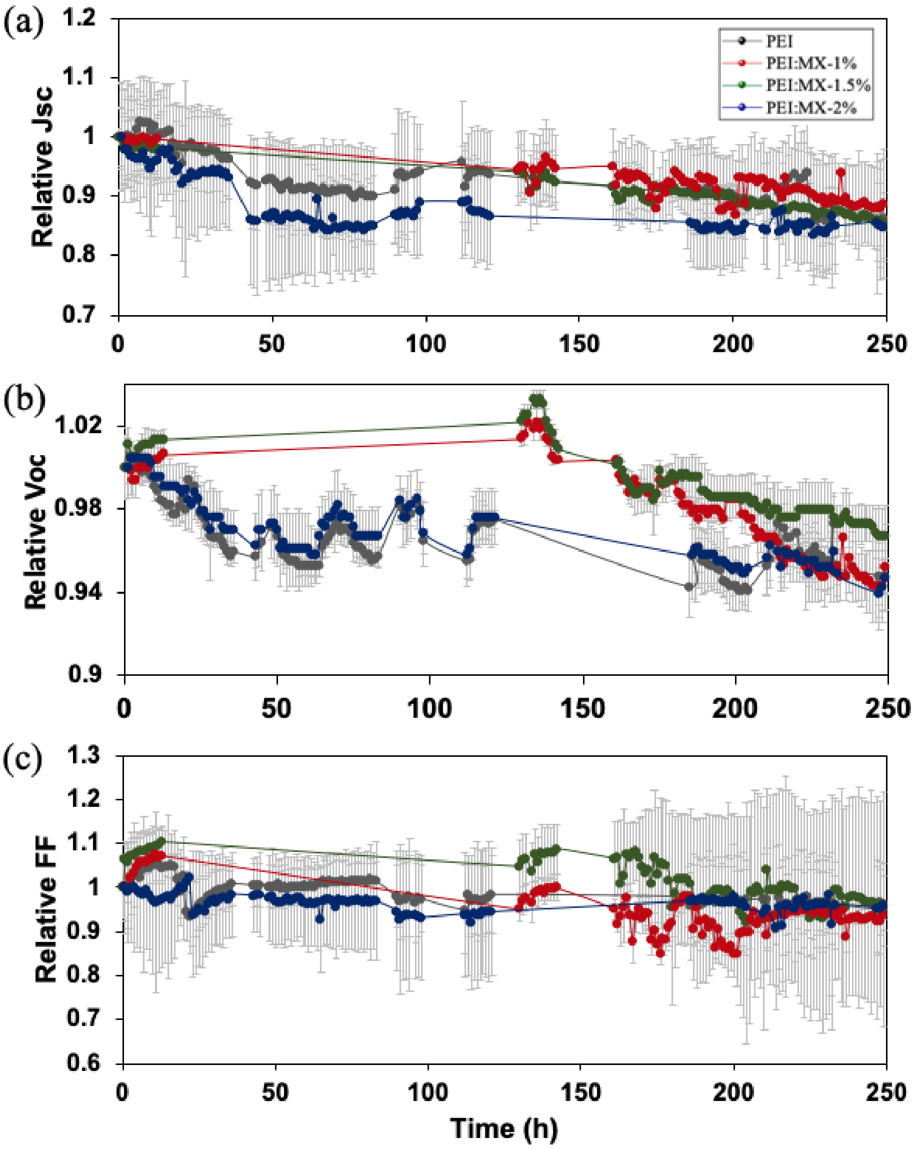

Figure S14: Normalized Jsc, Voc and FF-time curves for PEI and PEI/MX (1, 1.5 and 2%) obtained by ISOS-L-2 stability tests.

References

(1) Assunção, J. P. F.; Lemos, H. G.; Rossato, J. H. H.; Nogueira, G. L.; Lima, J. V. M.; Fernandes, S. L.; Nishihora, R. K.; Fernandes, R. V; Lourenço, S. A.; Bagnis, D.; et al. Interface Passivation with Ti3C2Tx-MXene Doped PMMA Film for Highly Efficient and Stable Inverted Perovskite Solar Cells. *J. Mater. Chem. C* **2024**, *12* (2), 562–574. https://doi.org/10.1039/D3TC03810F.

(2) Almora, O.; Aranda, C.; Mas-Marzá, E.; Garcia-Belmonte, G. On Mott-Schottky Analysis Interpretation of Capacitance Measurements in Organometal Perovskite Solar

Cells. *Appl. Phys. Lett.* **2016**, *109* (17), 173903. https://doi.org/10.1063/1.4966127.

(3) Almora, O.; García-Batlle, M.; Garcia-Belmonte, G. Utilization of Temperature-Sweeping Capacitive Techniques to Evaluate Band Gap Defect Densities in Photovoltaic Perovskites. *J. Phys. Chem. Lett.* **2019**, *10* (13), 3661–3669. https://doi.org/10.1021/acs.jpclett.9b00601.
